# Stem cell expression of CXCR4 regulates tissue composition in the vomeronasal organ

**DOI:** 10.1242/jcs.263451

**Published:** 2025-01-09

**Authors:** André Dietz, Katja Senf, Eva M. Neuhaus

**Affiliations:** Pharmacology and Toxicology, Jena University Hospital, Friedrich Schiller University Jena, Drackendorfer Str. 1, 07747 Jena, Germany

**Keywords:** Vomeronasal organ, Olfactory, Neurogenesis, VNO, CXCR4, Stem cell, Transcription factor, CellOracle

## Abstract

The vomeronasal organ (VNO) detects signaling molecules that often prompt innate behaviors, such as aggression and reproduction. Vomeronasal sensory neurons, classified into apical and basal lineages based on receptor expression, have a limited lifespan and are continuously replaced from a common stem cell niche. Using a combination of single-cell RNA sequencing data, immunofluorescence staining and lineage tracing, we identified CXCR4 expression in proliferative stem cells and the basal neuronal lineage. Mice with a conditional knockout of *Cxcr4* showed an increased number of SOX2-positive proliferative stem cells and enhanced basal neuronal lineage maturation. In addition, computational gene perturbation analysis revealed 87 transcription factors that might contribute to neurogenesis, among which was SOX2. Conditional knockout of *Cxcr4* did not only disturb neuronal maturation, but also affected non-neuronal cell types, resulting in a decrease of basal lamina lining quiescent stem cells and an increase in sustentacular support cells. Together, these findings enhance our understanding how a common pool of stem cells can give rise to different cell types of the VNO, highlighting the distinct role of CXCR4 in this process.

## Teaser

**Summary:** Expression of the chemokine receptor CXCR4 in stem cells of the vomeronasal organ is required for neurogenesis and for the maintenance of non-neuronal glia cells.

## INTRODUCTION

The vomeronasal organ (VNO) plays a pivotal role in detecting non-volatile molecules, which are essential for intra- and inter-species communication and the modulation of behaviors such as aggression and reproduction (Chamero et al., 2012; Holy et al., 2000; Papes et al., 2010; Stowers and Kuo, 2015). In the adult VNO, vomeronasal sensory neurons (VSNs) undergo continuous regeneration from a specialized stem cell niche located in the marginal zone of the organ (Brann and Firestein, 2010; Giacobini et al., 2000). Proper regulation of this niche is essential for maintaining the physiological distribution of various cell types ensuring the functionality of the VNO (Katreddi et al., 2022; Lin et al., 2022; Naik et al., 2020). Proliferating stem cells, termed globose basal cells, give rise to mature VSNs and sustentacular cells (Katreddi et al., 2022; LeFever et al., 2024). Neurogenesis in the VNO proceeds along distinct pathways, resulting in two main lineages of mature VSNs – apical VSNs and basal VSNs (Katreddi and Forni, 2021). These two lineages are distinguished by their localization within the sensory epithelium, and by the expression of different sets of vomeronasal receptors (Mombaerts, 2004; Pantages and Dulac, 2000).

Recent single-cell RNA sequencing studies have revealed a more detailed map of the cellular diversity within the VNO and the dynamic changes that occur during neurogenesis. These studies identified novel marker genes associated with different stages of VSN development and highlighted the crucial role of Notch1 signaling for the differentiation of neuronal precursors into basal VSNs (Katreddi et al., 2022). Additionally, the basal lineage-specific transcription factor AP2ε, encoded by transcription factor AP-2 epsilon (*Tfap2e*), has been shown to be crucial for basal VSN neurogenesis. Knockout of *Tfap2e* perturbs differentiation of neuronal progenitors into basal VSNs and diminishes the population of mature basal VSNs (Lin et al., 2018).

The G protein-coupled receptor C-X-C chemokine receptor 4 (CXCR4) is expressed in various cell types, encompassing stem cells, neurons, endothelial cells and hematopoietic cells, and thereby orchestrates diverse cellular functions, such as cell migration, homing, proliferation and neurogenesis (Molyneaux et al., 2003; Ratajczak et al., 2006; Tran et al., 2007; Zou et al., 1998). CXCR4 expression in globose basal cells and immature neurons of the main olfactory epithelium is required for proper neurogenesis. Knockout of CXCR4 in the olfactory epithelium enhances differentiation of globose basal cells and increases the number of mature olfactory sensory neurons (Senf et al., 2021). Moreover, exact adjustment of CXCL12 concentration in the olfactory epithelium via sustentacular cells is needed to establish a precise balance of CXCR4 signaling and thereby regulates neurogenesis (Dietz et al., 2024; Senf et al., 2021).

In the present study, we show that CXCR4 is also expressed by vomeronasal globose basal cells and immature basal VSNs, and plays a role in determining the cellular composition of the adult VNO. Utilizing a combination of bio-computational analysis, immunofluorescence experiments and quantitative PCR, we demonstrate that conditional knockout (cKO) of *Cxcr4* in vomeronasal globose basal cells results in enhanced basal VSNs generation. Furthermore, *Cxcr4* cKO impacts the replicative activity of sustentacular cells and leads to the absence of keratin 5 (KRT5)-expressing horizontal basal cells. These findings demonstrate the significance of CXCR4 signaling in VNO cell dynamics, highlighting its potential as a regulatory target in VNO function.

## RESULTS

### CXCR4 is expressed in globose basal cells and immature basal VSNs

Neurogenesis of the vomeronasal sensory epithelium occurs at the outer edges of the epithelium, where it interfaces with the non-sensory epithelium (Giacobini et al., 2000). This region, known as the marginal zone, serves as site of differentiation and proliferation of globose basal cells, the proliferative stem cells of the vomeronasal sensory epithelium. Globose basal cells give rise to intermediate neuronal cell stages and finally basal and apical VSNs (Fig. S1) (Katreddi and Forni, 2021). The sensory epithelium also comprises non-neuronal cells, such as horizontal basal cells and sustentacular cells; the non-sensory epithelium is composed of apical columnar cells and basal cells (Fig. S1) (LeFever et al., 2024).

**Fig. 1.**
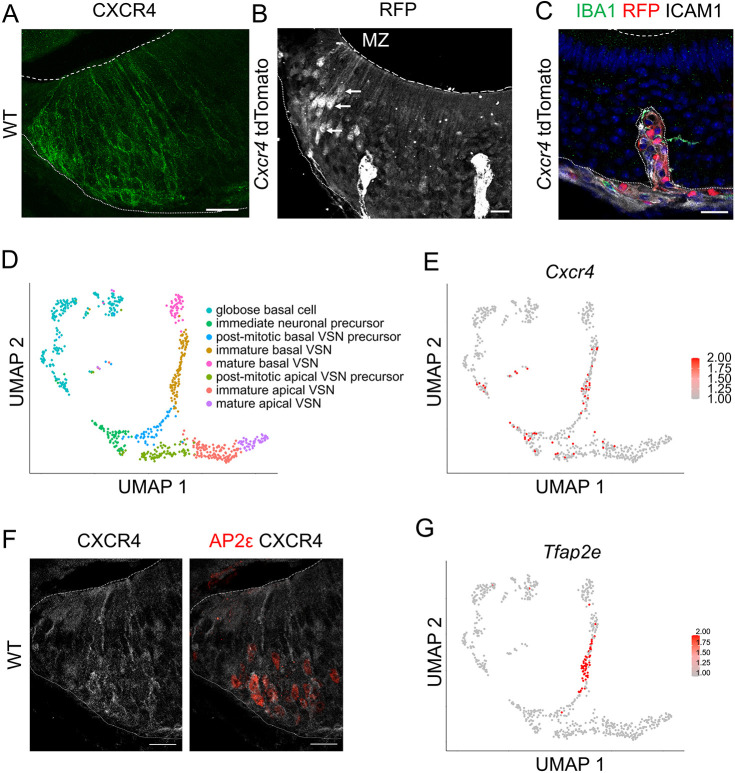
**CXCR4 is expressed in cells of the neuronal lineage within the marginal zone.** (A) Immunofluorescence staining of CXCR4 in the VNO of WT mice (8 weeks old) shows that it is expressed in cells of the marginal zone. (B) Immunofluorescence staining of mice expressing the red fluorescence protein (RFP) tdTomato under control of the *Cxcr4* promoter shows activation of *Cxcr4* transcription in cells of the marginal zone (MZ) and in maturing sensory neurons, identified by neuronal dendrites and knobs (indicated by arrows). (C) Co-staining of mice expressing the red fluorescence protein tdTomato (red) under control of the *Cxcr4* promoter, ICAM1, labeling blood vessels (white), and IBA1, labeling macrophages (green), showing expression of *Cxcr4* in both structures. Images in A–C are representative of five or six repeats. (D) Reproduction of neuronal subset UMAP reduction plot from the scRNAseq dataset GSE190330 (Katreddi et al., 2022). Clustering shows differentiation of globose basal cells into immediate neuronal precursors before splitting up into apical and basal immature neuronal lineage. (E) Feature plot visualization of *Cxcr4* gene expression per cell shows expression in globose basal cells and immature basal VSNs. (F) Immunofluorescence staining shows co-expression of the basal lineage marker protein AP2ε (red) and CXCR4 (white) in WT mice. Images representative of three repeats. (G) Feature plot visualization of *Tfap2e* (encoding AP2ε) gene expression per cell shows expression in immature basal VSNs. In images, fine dotted lines represent the basal limitation, dashed lines the apical limitation of the VNO. Scale bars 20 µm.

Immunofluorescence experiments of the VNO revealed localization of CXCR4 within cells of the marginal zone (Fig. 1A). Given its spatial distribution, CXCR4 is likely expressed by cells engaged in the process of neurogenesis. Additional experiments, utilizing mice expressing the fluorescent protein tdTomato under control of the *Cxcr4* promoter, validated the expression of *Cxcr4* within the marginal zone of the VNO (Fig. 1B). The reporter was also expressed in other structures, which were identified as intercellular adhesion molecule 1 (ICAM1)-positive blood vessels and ionized calcium-binding adapter molecule 1 (IBA1, also known as AIF1)-positive macrophages (Fig. 1C; Fig. S2A), as expected from the well-known expression of *Cxcr4* in both structures (Salvucci et al., 2002; Werner et al., 2020). Absence of clear antibody staining for CXCR4 in these structures is likely due to low expression levels. Detailed analysis revealed tdTomato expression in lateral cells showing a neuronal morphology (indicated by arrows in Fig. 1B), further confirming *Cxcr4* expression in the neuronal lineage. VSNs within the medial part of the VNO did not show clear tdTomato expression. *Cxcr4*-driven tdTomato expression was induced by Tamoxifen application in mice aged 3 weeks. Missing tdTomato expression therefore indicates that medial neurons are not replaced within a time frame of 5 weeks. VNO neurogenesis is known to proceed horizontally from the marginal zone towards the medial region (Giacobini et al., 2000; Martinez-Marcos et al., 2005), mature medially localized neurons therefore have to move from the marginal zone through the epithelium before the appearance in the medial part of the epithelium.

Reanalysis of a publicly available single cell RNA sequencing (scRNAseq) dataset of the VNO, specifically subsetted to track neurogenesis from stem cells to maturing neurons, delineates several intermediate cell states (Katreddi et al., 2022). These include proliferative stem cells (globose basal cells), immediate neuronal precursors, basal and apical post-mitotic VSN precursors, basal and apical immature VSNs and mature VSNs (Fig. 1D). Visualization of the scRNAseq dataset for *Cxcr4* expression within the neuronal lineage of the VNO demonstrates *Cxcr4* expression predominantly in globose basal cells, immediate neuronal progenitor cells and basal immature VSNs (Fig. 1E). Co-immunofluorescence staining against CXCR4 and AP2ε, a transcription factor known to be expressed in basal immature VSNs (Lin et al., 2018), reveals colocalization of both proteins in the same cell type (Fig. 1F). Feature plot visualization of *Tfap2e* within the neuronal scRNAseq subset shows expression of *Tfap2e* in basal immature VSNs of the VNO, similar to what is seen for *Cxcr4* (Fig. 1E), supporting the finding of co-expression of both proteins (Fig. 1G). Examination of a distinct web-based scRNAseq dataset, also discriminating cells of the non-sensory epithelium (Gvs et al., 2024), revealed the expression of the CXCR4 ligand C-X-C motif chemokine 12 (CXCL12) by sustentacular cells and basal cells of the non-sensory epithelium. Interestingly, the CXCL12 scavenging receptor atypical chemokine receptor 3 (ACKR3) shows co-expression within the same cells (Fig. S2B), and might serve as a regulator of CXCL12 concentration within the VNO as already shown for the main olfactory epithelium (Dietz et al., 2024).

In conclusion, our findings identified distinct expression patterns of *Cxcr4* in the marginal zone across various stages of VSN development and lineage tracing suggests its involvement in neurogenesis. Co-expression with *Tfap2e* further suggests a role of CXCR4 specifically in the basal lineage of VSNs.

### Loss of CXCR4 signaling disturbs differentiation of globose basal cell in the VNO

**Fig. 2.**
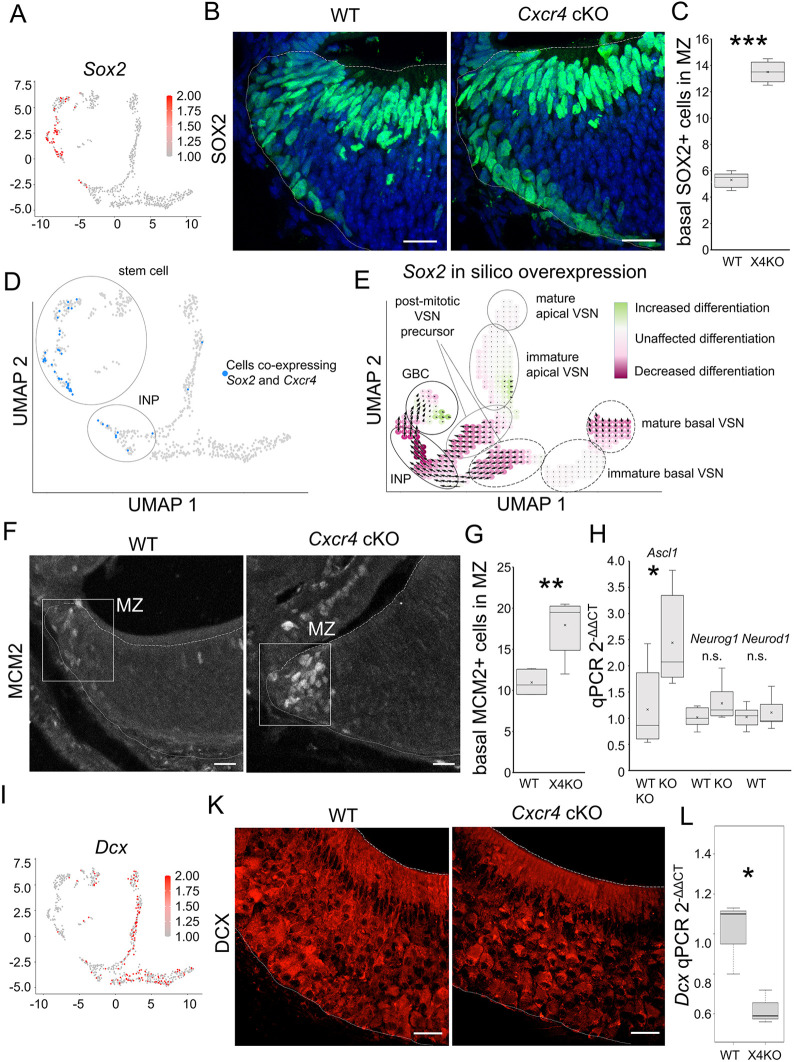
**Globose basal cell-specific knockout of *Cxcr4* impairs neuronal differentiation.** (A) Visualization of cells expressing *Sox2* within the neuronal scRNAseq subset shows localization in stem cells and immediate neuronal progenitor cells. (B) Immunofluorescence staining of SOX2 (green) in WT and *Cxcr4* cKO mice shows localization in globose basal cells within the marginal zone, nuclei are stained with Hoechst (blue). (C) Quantification of basally located SOX2-expressing cells within the marginal zone showing a significant increase in number of globose basal cells in *Cxcr4* cKO (X4KO) mice (*n*=5 animals per group). ***P<0.001 (two-tailed unpaired Student's *t*-test). (D) Visualization of cells co-expressing *Sox2* and *Cxcr4* within the neuronal scRNAseq subset shows localization in stem cells and immediate neuronal progenitors. (E) CellOracle *Sox2* computational overexpression analysis of the neuronal scRNAseq subset shows the strongest decrease in differentiation of cells transitioning from globose basal cells (GBC) into immediate neuronal progenitors (INP). (F) Immunofluorescence staining of MCM2 in WT and *Cxcr4* cKO mice shows localization of replicating globose basal cells within the marginal zone. (G) Quantification of the number of MCM2-expressing globose basal cells within the marginal zone showing a significant increase in *Cxcr4* cKO mice (*n*=5 animals per group). **P<0.01 (two-tailed unpaired Student's *t*-test). (H) Quantification of mRNA transcripts by qPCR showing a significant increase of expression of *Ascl1* in *Cxcr4* cKO (X4), increases in *Neurog1* and *Neurod1* expression were not significant (*n*=6 animals per group. *P<0.05; ns, not significant (two-tailed unpaired Student's *t*-test). (I) Visualization of cells expressing *Dcx* within the neuronal scRNAseq subset shows localization in immature and mature VSNs. (K) Immunofluorescence staining of DCX in WT and *Cxcr4* cKO mice shows decreased fluorescence intensity in *Cxcr4* cKO mice. (L) Quantification of *Dcx* mRNA transcripts by qPCR showing a significant decrease of *Dcx* expression in *Cxcr4* cKO mice (*n*=3 animals per group). *P<0.05 (two-tailed unpaired Student's *t*-test). Fine dotted lines represent the basal limitation, dashed lines the apical limitation of the VNO. *P<0.05. For box plots in C, G, H and L, the box represents the 25–75th percentiles, and the median (line) and mean (cross) are indicated. The whiskers show the minimum and maximum values of the dataset. Scale bars: 20 µm.

To investigate the potential regulatory role of CXCR4 signaling in vomeronasal neurogenesis, we utilized mice with a conditional knockout of *Cxcr4* (*Cxcr4* cKO) under the control of the 5-hydroxytryptamine receptor 3A (*Htr3a*) promoter (*Htr3a*-Cre*;Cxcr4*^LoxP/LoxP^). Reporter strains of mice expressing green fluorescent protein (GFP) under the control of the *Htr3a* promoter revealed *Htr3a* expression in globose basal cells (Finger et al., 2017). Given our observation that *Cxcr4* expression initiates in globose basal cells, we examined the distribution of the globose basal cell marker sex determining region Y-box 2 (SOX2) (Fig. 2A), a transcription factor indicating cellular stemness (LeFever et al., 2024). Immunofluorescence experiments demonstrated a 2.5-fold increase in number of SOX2-expressing globose basal cells within the marginal zone, which can be clearly distinguished from apical sustentacular cells of *Cxcr4* cKO mice (Fig. 2A–C). Feature plot visualization of the neuronal lineage scRNAseq data revealed the co-expression of *Sox2* and *Cxcr4* in late globose basal cells and early immediate neuronal progenitor cells (Fig. 2D).

To examine the function of *Sox2* expression within the gene regulatory network of VNO neurogenesis, we applied CellOracle analysis, a Python-based computational method combining pseudotime analysis with gene perturbation analysis, to the scRNAseq dataset of the neuronal lineage (Kamimoto et al., 2023). Computational overexpression of *Sox2* showed inhibition of globose basal cell differentiation, validating the necessity of declining *Sox2* expression for sufficient differentiation from globose basal cells into immediate neuronal progenitors, particularly at the transition point where *Sox2* is co-expressed with *Cxcr4* (Fig. 2E). Moreover, *Cxcr4* cKO mice showed a significant increase in minichromosomal maintenance 2 (MCM2)-expressing cells within the marginal zone. As MCM2 is a proliferation marker initiating DNA replication, this supports the findings of increased number of proliferative globose basal cells (Fig. 2F,G) (Kelly and Brown, 2000). The Cre driver line (*Htr3a*-Cre) did not show increased MCM2 or SOX2 staining (Fig. S3B). Only few cells co-express SOX2 and MCM2 (Fig. S3B), which is expected given that high expression levels of SOX2 maintains stem cells of the central nervous system in a slowly self-renewing, undifferentiated state, and proliferation is accompanied by decreased SOX2 expression (Hagey and Muhr, 2014).

Quantitative PCR analysis of neurogenic basic-helix-loop-helix (bHLH) family transcription factors that are essential for the specification and differentiation of the neurogenic progenitors revealed increased expression of achaete-scute homolog 1 (*Ascl1*), whereas neurogenic differentiation 1 (*Neurod1*) and neurogenin 1 (*Neurog1*) were slightly increased, but not significantly altered (Fig. 2H). *Ascl1* is predominantly expressed by neuronal progenitors (Murray et al., 2003), which further divide and give rise to immediate neuronal precursors expressing *Neurog1* and *Neurod1* (Hills et al., 2024; Katreddi and Forni, 2021). To follow neuronal differentiation in the VNO, we used immunofluorescence staining of doublecortin (DCX), a marker for developing neurons (Brown et al., 2003) (Fig. 2I; Fig. S3C,D), and observed a decline in DCX expression in *Cxcr4* cKO mice compared to that in wild-type (WT) mice (Fig. 2K). Quantitative PCR analysis moreover showed a significant decrease in *Dcx* mRNA expression in *Cxcr4* cKO mice (Fig. 2L). Immunofluorescence experiments of *Cxcr4* cKO mice and computational analysis collectively suggest that CXCR4 plays a role in regulating stemness and neuronal differentiation within the VNO.

### Enhanced neuronal differentiation of basal VSNs in *Cxcr4* cKO animals

**Fig. 3.**
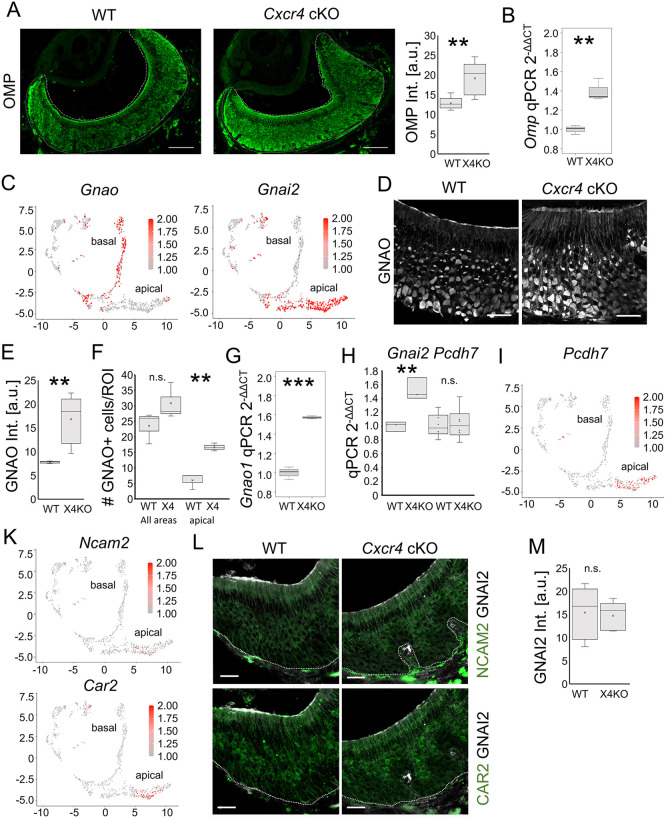
***Cxcr4* cKO leads to increase in basal neuronal population size.** (A) Immunofluorescence staining of OMP in WT and *Cxcr4* cKO mice shows an increase in fluorescence intensity and in basal VSN density in *Cxcr4* cKO mice. Quantification of OMP fluorescence intensity shows a significant increase in *Cxcr4* cKO mice (*n*=5 animals per group), **P<0.01 (Welch test). (B) qPCR showing a significant increase of *Omp* expression in *Cxcr4* cKO (X4KO) mice (*n*=3 animals per group) **P<0.01 (two-tailed unpaired Student's *t*-test). (C) Visualization of cells expressing *Gnao* and *Gnai2* within the neuronal scRNAseq subset shows localization in basal immature and mature VSNs. (D) Immunofluorescence staining of GNAO in WT and *Cxcr4* cKO mice. (E) Quantification of GNAO fluorescence intensity shows a significant increase in *Cxcr4* cKO mice (*n*=5 animals per group). **P<0.01 (two-tailed unpaired Student's *t*-test). (F) Quantification of GNAO-positive cells shows a significant increase in the apical region of the epithelium in *Cxcr4* cKO mice, whereas the total number of GNAO-positive cells was not different (*n*=3 animals per group). **P<0.01; n.s., not significant (two-tailed unpaired Student's *t*-test). (G) Quantification of *Gnao1* mRNA transcripts by qPCR showing a significant increase of *Gnao1* expression in *Cxcr4* cKO mice (*n*=3 animals per group). ***P<0.001 (two-tailed unpaired Student's *t*-test). (H) Quantification of *Gnai2* and *Pcdh7* mRNA transcripts by qPCR shows a significant increase of *Gnai2* expression due to expression of *Gnai2* in multiple cell types of the VNO, but no difference in expression of *Pcdh7*, which is specifically expressed by apical VSNs (*n*=6 animals per group). **P<0.01; n.s., not significant (two-tailed unpaired Student's *t*-test). (I) Visualization of cells expressing *Pcdh7* within the neuronal scRNAseq subset shows localization in apical mature VSNs. (K) Visualization of cells expressing *Car2* and *Ncam2* within the neuronal scRNAseq subset shows localization in apical immature and mature VSNs. (L) Immunofluorescence staining of WT and *Cxcr4* cKO mice with the apical VSN markers GNAI2 (white) together with NCAM2 or CAR2 (green) showing no difference in apical mVSN population size. Images representative of three repeats. (M) Quantification of GNAI2 fluorescence intensity shows no significant difference between WT and *Cxcr4* cKO mice (*n*=5 animals per group n.s., not significant (two-tailed unpaired Student's *t*-test). Fine dotted lines represent the basal limitation, dashed lines the apical limitation of the VNO. For box plots in A, B, E–H and M, the box represents the 25–75th percentiles, and the median (line) and mean (cross) are indicated. The whiskers show the minimum and maximum values of the dataset. Scale bars: 100 µm (A), 20 µm (D,L). a.u., arbitrary units.

Given the perturbation in stem cell differentiation resulting from the absence of *Cxcr4*, we conducted further investigations into the impact of *Cxcr4* cKO on the distribution of mature VSNs. Analysis of fluorescence intensities of immunofluorescence staining and mRNA expression via qPCR showed a significant increase in the expression of olfactory marker protein (OMP), a specific marker for mature neurons in the VNO (Fig. S4A,B), at the protein (Fig. 3A) and transcript level (Fig. 3B), in *Cxcr4* cKO mice. Notably, especially the basal part of the vomeronasal sensory epithelium showed higher fluorescence intensities and fewer non-labeled areas in *Cxcr4* cKO mice, indicating a denser packaging of mature VSNs (Fig. 3A).

Next, we investigated both lineages of VSNs, characterized by expression of specific G protein subunits for signal transduction (Jia and Halpern, 1996). VSNs of the basal lineage of the vomeronasal sensory epithelium express the heterotrimeric G protein subunit *Gnao*, whereas neurons of the apical lineage express *Gnai2* (Fig. 3C). Specific identification of basal VSNs through staining of GNAO demonstrated a significant increase in the fluorescence intensity in *Cxcr4* cKO mice (Fig. 3D,E; Fig. S4C). The effect was caused by absence of *Cxcr4*, as the Cre driver line (*Htr3a*-Cre) did not show increased GNAO staining (Fig. S4D). Moreover, the basal population of VSNs expanded into the apical part of the vomeronasal sensory epithelium, as we found that the relative number of GNAO-positive cells was significantly different between genotypes only in the apical part of the epithelium (Fig. 3D,F). Also mRNA levels of *Gnao* were significantly increased (Fig. 3G). These findings suggest an enhanced differentiation of basal immature VSNs under *Cxcr4* cKO conditions.

Expression of *Gnai2* mRNA was also increased under *Cxcr4* cKO conditions (Fig. 3H). However, although *Gnao* is specifically expressed by neurons of the VNO, *Gnai2* is expressed by apical neurons and by epithelial cells, immune cells and pericytes (Fig. S4E,F). The cell type expressing the gene is unclear due to the broad expression pattern. To identify additional markers for apical VSNs, we performed differential expression analysis of genes expressed by mature apical VSNs compared to all other cell types. This approach revealed *Car2*, *Ncam2* and *Pcdh7* as specific markers for the apical lineage (Fig. 3I,K). Expression of the apical lineage specific marker gene *Pcdh7* did not reveal significant differences (Fig. 3H). Moreover, localization and expression levels of GNAI2, NCAM2 and CAR2 were not different between WT and *Cxcr4* cKO mice (Fig. 3L,M; Fig. S4C). In summary, the observed increase in OMP levels, along with the expansion of the basal VSN population reveals the regulatory role of CXCR4 in maturation of the basal neuronal lineage.

### Computational identification of transcription factors affecting VNO neurogenesis

**Fig. 4.**
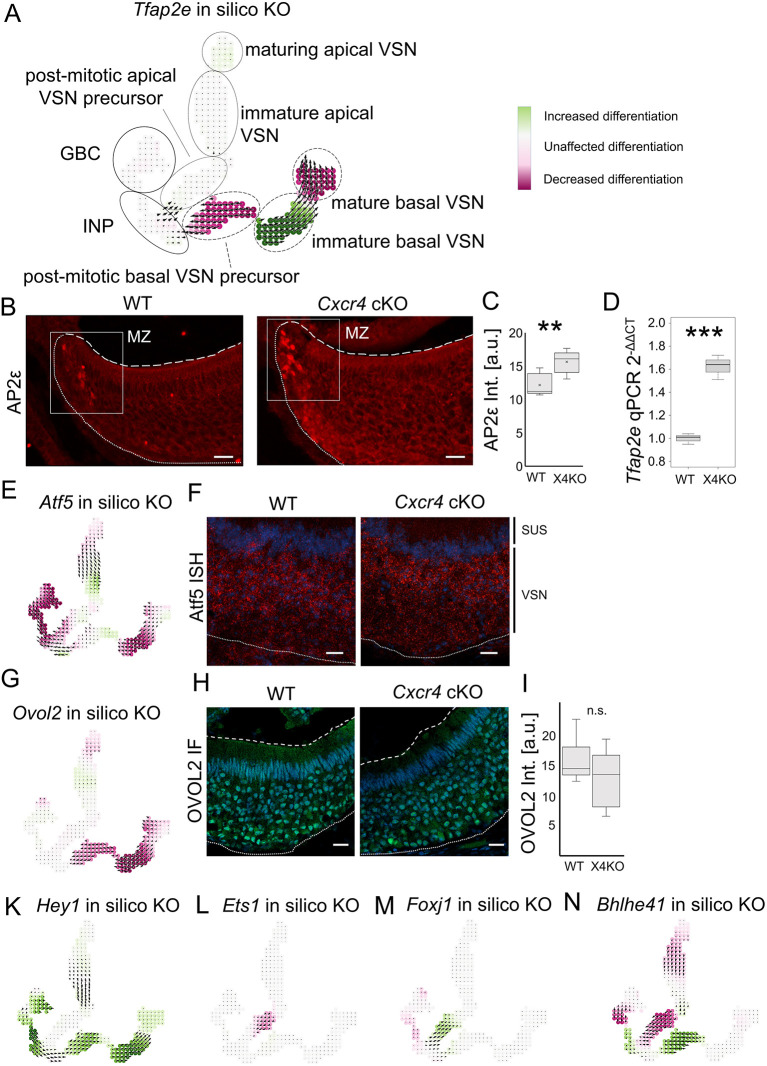
**Computational identification of transcription factors putatively altering lineage-specific neurogenesis.** (A) CellOracle computational knockout analysis of *Tfap2e* verifies the used approach to correctly predict decreased differentiation within the basal neuronal lineage. Vector maps show transitions in cell identity, color codes show the directionality of the perturbation vector to the natural differentiation vector. Positive perturbation scores (green) indicate that reduced expression of these transcription factors promotes differentiation. Negative perturbation scores (purple) show reduced differentiation. Solid circles highlight multipotent stem cells, dotted circles highlight the apical neuronal lineage, and dashed circles highlight the basal neuronal lineage. (B) Immunofluorescence staining of AP2ε in WT and *Cxcr4* cKO (X4KO) mice. (C) Quantification of AP2ε fluorescence intensity within the marginal zone showing a significant increase in *Cxcr4* cKO mice (*n*=5 animals per group). **P<0.01 (two-tailed unpaired Student's *t*-test). (D) Quantification of *Tfap2e* mRNA transcripts by qPCR showing a significant increase of *Tfap2e* expression in *Cxcr4* cKO mice (*n*=3 animals per group). ***P<0.001 (two-tailed unpaired Student's *t*-test). (E) *Atf5* shows an inhibitory effect on basal lineage differentiation. (F) *In situ* hybridization of *Atf5* in the VNO of WT and Cxcr4 cKO mice shows expression of *Atf5* (red) mostly in the apical neurons, nuclei are labeled with Hoechst (blue). sus, sustentacular cells. (G) *Ovol2* shows a specific inhibitory effect on basal lineage differentiation. (H) OVOL2 is expressed by all neurons in the VNO without marked lineage specificity. (I) Quantification of OVOL2 fluorescence intensity within the basal epithelium showing no difference between genotypes (*n*=3 animals per group). n.s., not significant (two-tailed unpaired Student's *t*-test). (K) *Hey1* has a strong enhancing effect on basal lineage differentiation. (L,M) *Ets1* shows an inhibitory (L) and *Foxj1* shows an enhanced effect (M) on apical lineage differentiation. (N) Bhlhe41 shows an increase in basal lineage differentiation and an inhibitory effect on apical lineage differentiation. Fine dotted lines represent the basal limitation, dashed lines the apical limitation of the VNO. For box plots in C, D and I, the box represents the 25–75th percentiles, and the median (line) and mean (cross) are indicated. The whiskers show the minimum and maximum values of the dataset. Scale bars 20 µm. a.u., arbitrary units.

Absence of CXCR4 has an effect on the dichotomy of the VNO and causes increased numbers of basal lineage VSNs. The transcription factor *Tfap2e* has been described to affect the basal neuronal differentiation or maturation program, and loss of AP-2ε in KO mice induces a progressive loss of basal VSNs (Lin et al., 2018). Computational perturbation analysis for *Tfape2* using CellOracle indicated that this gene has an inhibitory effect on maturation of basal VSNs (Fig. 4A), demonstrating that CellOracle is capable of predicting the correct outcome. In agreement with the observed increase in the number of basal VSNs, immunofluorescence experiments against AP2ε revealed an increase in fluorescence intensity of basal immature VSN in *Cxcr4* cKO mice (Fig. 4B,C), along with a significant increase of *Tfap2e* expression (Fig. 4D).

Identification of other transcription factors that can control neuronal fate could provide relevant insights into CXCR4-dependent lineage specification. Owing the power of the CellOracle approach for prediction of transcription factor effects on VNO neurogenesis, we performed a computational perturbation to screen all transcription factors expressed in the VNO and found several factors with a putative role in differentially influencing lineage fate (Fig. S5).

Knockout of *Atf5* has been shown to reduce the differentiation, survival and axonal projection of basal VSNs (Nakano et al., 2016). Again, CellOracle analysis of *Atf5* KO revealed inhibition of basal VSNs (Fig. 4E). *Atf5* mRNA is strongly expressed by all VSNs during the neonatal stage (Nakano et al., 2016), whereas we found postnatal expression enriched in the apical neuronal layer (Fig. 4F). However, *Atf5* might not be linked to *Cxcr4* expression, as *in situ* hybridization of *Atf5* revealed comparable expression levels in *Cxcr4* cKO and WT mice (Fig. 4F). Another transcription factor showing a strong predicted effect on inhibition of basal VSN neurogenesis was *Ovol2* (Fig. 4G). OVOL2 was specifically expressed by VSNs, given that we did not detect OVOL2 in sustentacular cells (Fig. 4H). Expression was not apparently lineage specific but detected in all VSNs, without marked differences between genotypes (Fig. 4H,I). OVOL2 is involved in the maintenance of epithelial identity and is known to inhibit LSD1-mediated H3K4me2 demethylation to activate epithelial genes (Zhong et al., 2023). Although not described in the VNO yet, LSD1 is required for maturation of sensory neurons of the main olfactory system (Coleman et al., 2017). Other transcription factors with marked inhibitory effects especially in immature basal VSNs were *Batf3* and *Prrxl1* (Fig. S5).

Proteins that enhanced the proliferative effects on the basal lineage were *Hey1* (Fig. 4K), and at various strengths, *Bach1*, *E2f1*, *Hes5*, *Pou3f1* and *Sox11* (Fig. S5). Apical lineage exclusive effects were identified for the transcription factor *Ets1*, inhibiting apical neurogenesis, and *Foxj1*, promoting apical neurogenesis (Fig. 4L,M). A promoting effect specific for the apical lineage along with stronger effects on stem cell differentiation was seen for *Elf3*, *Six3* and *Stat1* (Fig. S5). Transcription factors showing effects on both lineages often predicted inhibition of the apical lineage along with enhancing effects on the basal lineage, namely *Bhlhe41* (Fig. 4N), *Gabpa*, *Pbx3*, *Egr1*, *Klf2*, *Klf5* and *Tcfl5* (Fig. S5). Inhibition of the basal lineage and promotion of the apical lineage was identified for *Klf3*, *Klf6* and *Nrf1* (Fig. S5). Altogether, 87 of 134 tested transcription factors showed an impact on neurogenesis in CellOracle perturbation analysis (Fig. S5), and we identified 19 targets showing strong lineage specific effects (*Bach1*, *Batf3*, *Bhlhe41*, *E2f1*, *Egr1*, *Gabpa*, *Hes5*, *Hey1*, *Klf2*, *Klf3*, *Klf5*, *Klf6*, *Nrf1*, *Ovol2*, *Pbx3*, *Pou3f1*, *Prrxl1*, *Sox11* and *Tcfl5*) for future analysis.

### Absence of CXCR4 alters abundance of horizontal basal cells and sustentacular cells

**Fig. 5.**
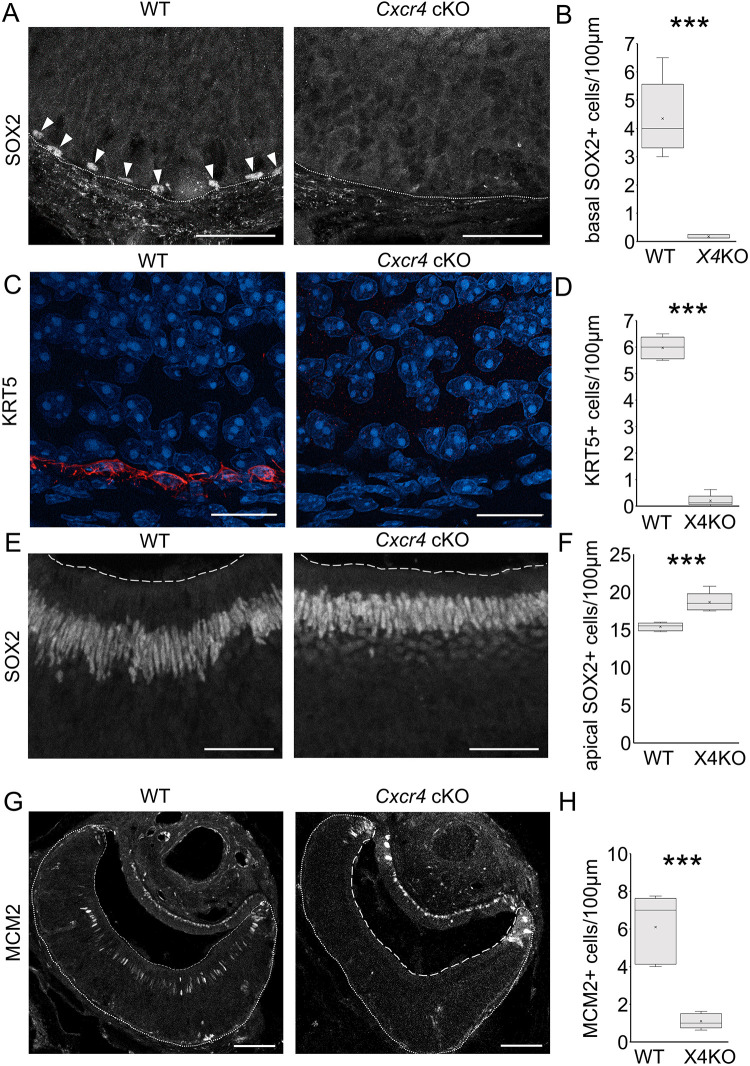
***Cxcr4* cKO leads to depletion of horizontal basal cells and increase in number of sustentacular cells.** (A) Immunofluorescence staining of SOX2 in WT and *Cxcr4* cKO (X4KO) mice shows almost complete loss of horizontal basal cell staining in *Cxcr4* cKO mice. White arrowheads highlight location of SOX2-expressing horizontal basal cells. (B) Quantification of SOX2-expressing horizontal basal cells showing a significant decrease in *Cxcr4* cKO mice (*n*=5 animals per group). ***P<0.001 (two-tailed unpaired Student's *t*-test). (C) Immunofluorescence staining of KRT5 in WT and *Cxcr4* cKO mice shows complete loss of horizontal basal cells in the medial area of *Cxcr4* cKO mice. (D) Quantification of KRT5-expressing horizontal basal cells showing a significant decrease in *Cxcr4* cKO mice (*n*=5 animals per group). ***P<0.001 (two-tailed unpaired Student's *t*-test). (E) Immunofluorescence staining of SOX2 in WT and *Cxcr4* cKO mice shows increased abundance of sustentacular cells in *Cxcr4* cKO mice. (F) Quantification of SOX2-expressing sustentacular cells showing a significant increase in *Cxcr4* cKO mice (*n*=5 animals per group). ***P<0.001 (two-tailed unpaired Student's *t*-test). (G) Immunofluorescence staining of MCM2 in WT and *Cxcr4* cKO mice shows almost complete loss of MCM2 expression in sustentacular cells of *Cxcr4* cKO mice. (H) Quantification of MCM2-expressing sustentacular cells showing a significant decrease in *Cxcr4* cKO mice (*n*=5 animals per group). ***P<0.001 (two-tailed unpaired Student's *t*-test). Fine dotted lines represent the basal limitation, dashed lines the apical limitation of the VNO. For box plots in B, D, F and H, the box represents the 25–75th percentiles, and the median (line) and mean (cross) are indicated. The whiskers show the minimum and maximum values of the dataset. Scale bars: 50 µm (A,E), 20 µm (C), 100 µm (G).

Further examination of the VNO revealed that the impact of *Cxcr4* cKO extends beyond neurogenesis and also affected non-neuronal cells, such as horizontal basal cells and sustentacular cells. Immunofluorescence experiments revealed expression of the stemness marker SOX2 in horizontal basal cells of WT mice, and an almost complete loss of SOX2 expression in horizontal basal cells of *Cxcr4* cKO mice (Fig. 5A,B). To elucidate whether the loss of SOX2 expression in horizontal basal cells is caused by a complete absence of horizontal basal cells or an alteration of horizontal basal cell stemness, we analyzed the localization of KRT5, which has been shown to be expressed in horizontal basal cells of the VNO (LeFever et al., 2024) (Fig. S6A,B). Immunofluorescence experiments demonstrated a significant decrease of KRT5 staining in *Cxcr4* cKO mice (Fig. 5C,D; Fig. S6C), providing evidence for a marked reduction in horizontal basal cells. Interestingly, immunofluorescence experiments against SOX2 showed a significant increase in number of sustentacular cells in *Cxcr4* cKO mice (Fig. 5E,F). Furthermore, staining of the DNA replication marker MCM2, also expressed by sustentacular cells in WT mice, revealed an almost complete loss of MCM2 staining in sustentacular cells of *Cxcr4* cKO mice (Fig. 5G,H), whereas MCM2 expression and cell proliferation was increased in the marginal zone containing the stem and precursor cells (Fig. 2F,G). In conclusion, the loss of SOX2 and KRT5 staining of horizontal basal cells in *Cxcr4* cKO mice indicates an effect of CXCR4 on horizontal basal cell maintenance or migration during development. Moreover, the increase in cell number of SOX2-expressing sustentacular cells in *Cxcr4* cKO mice also suggests a crucial role for CXCR4 in regulating proliferation of sustentacular cells.

## DISCUSSION

**Fig. 6.**
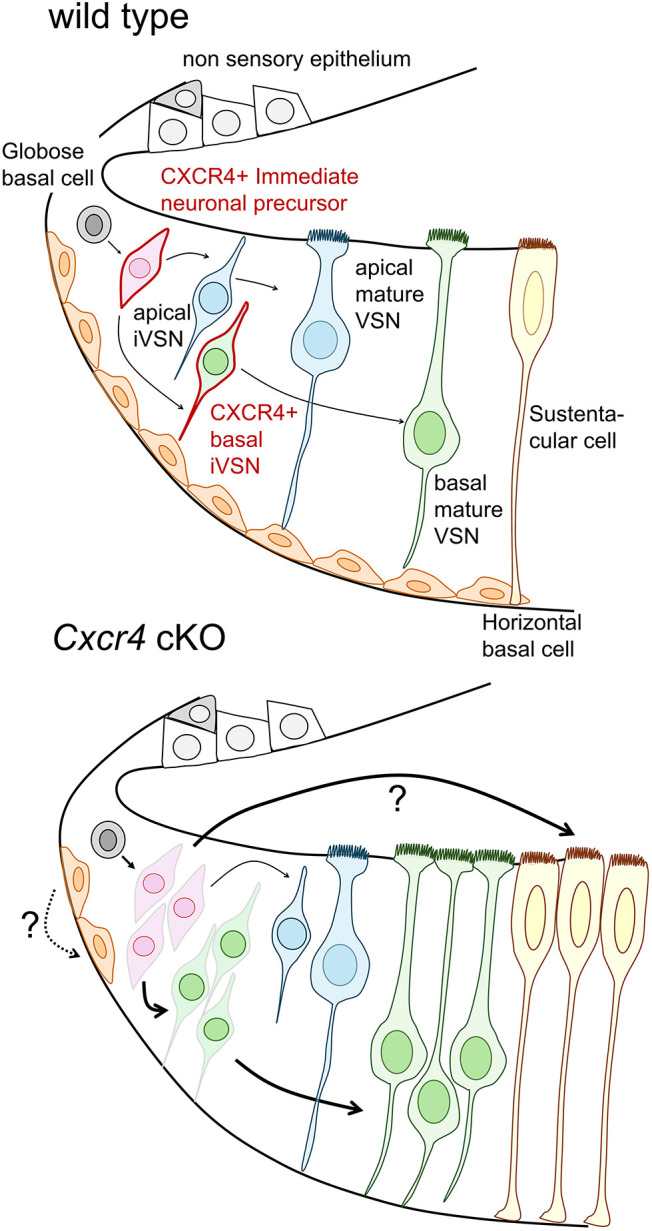
***Cxcr4* cKO affects different cell types in the vomeronasal organ.** Schematic illustration of the results. CXCR4 is expressed in globose basal cells and immature basal VSNs (outlined in red). *Cxcr4* cKO causes an increased proliferation of cells of the marginal zone (pink) and increases the abundance of VSNs of the basal lineage (green). Moreover, *Cxcr4* cKO alters non-neuronal cells, causing a reduction in the number of KRT5-expressing horizontal basal cells (orange) and an increase in SOX2-positive sustentacular cells (yellow).

The VNO relies on a precise balance between two subtypes of VSNs to ensure consistent social behaviors in mice (Chamero et al., 2012; Lin et al., 2022). The cellular identity of the apical and basal VSNs is known to be determined by the expression of lineage-specific transcription factors (Katreddi and Forni, 2021; Katreddi et al., 2022; Lin et al., 2018). Here, we demonstrate that the chemokine receptor CXCR4 is expressed in globose basal cells and neuronal progenitors and regulates neurogenesis within the VNO. *Cxcr4* cKO mice showed enhanced differentiation of basal VSNs and an alteration in number and proliferative status of sustentacular cells, along with an absence of horizontal basal cells in the medial part of the VNO (Fig. 6).

CXCR4 signaling regulates proper homeostasis of neurogenesis via its ligand CXCL12 within the main olfactory epithelium, leading to a decrease in immature neurons and an increase in mature neurons upon CXCR4 downregulation (Dietz et al., 2023; Senf et al., 2021). In this study our findings demonstrate a regulatory role of CXCR4 signaling in neurogenesis of the VNO. Specifically, the conditional KO of *Cxcr4* in globose basal cells of the VNO revealed a significant decrease in DCX expression in immediate neuronal progenitor cells and an increase in OMP expression in mature VSNs, mirroring the effects observed in the main olfactory epithelium. However, the underlying regulatory mechanisms of CXCR4 signaling might be different. Notably, we observed an increase in the number of MCM2-expressing globose basal cells in the VNO compared to a decrease in the number of globose basal cells within the main olfactory epithelium. The increase in number of SOX2-expressing globose basal cells together with a decrease in neuronal progenitors could indicate a stalling of early neuronal differentiation. By contrast, *Cxcr4* expression extends into the basal lineage of immature VSNs and maturation of basal VSN was increased upon *Cxcr4* knockout. Whether CXCR4 affects different stages of differentiation or whether the reduced amount of DCX-positive neuronal progenitors is a consequence of increased maturation of basal VSNs is unclear at the moment. Interestingly, the number of apical VSN remained unchanged, indicating that enhanced basal cell maturation might play a role.

Recent findings demonstrate that the lineage-specific expression of *Notch1* and delta-like 4 (*Dll4*) mediates fate decisions in the neuronal lineage of the VNO (Katreddi et al., 2022). Basal immature VSNs express *Notch1*, which has been shown to induce *Cxcr4* expression (Fabbri et al., 2017; Liu et al., 2019). In contrast, apical immature VSNs express *Dll4*, which has been shown to suppress *Cxcr4* expression (Williams et al., 2008). This supports our observation of lineage-specific expression of *Cxcr4*, suggesting that its transcriptional regulation could be mediated by the Notch pathway. Additionally, disruption of transforming growth factor β (TGFβ) and bone morphogenic protein (BMP) canonical signaling pathways via knockout of their transduction mediator SMAD4 has been shown to downregulate basal VSN development (Naik et al., 2020). Interestingly, loss of SMAD4 in pancreatic cancer cells has been shown to shift TGFβ-triggered downstream cascades to mitogen-activated protein kinase (MAPK) and extracellular signal-regulated kinase (ERK) signaling, a pathway also known to be triggered upon CXCR4 activation (Bianchi and Mezzapelle, 2020; Hong et al., 2009). CXCR4 might therefore regulate the MAPK/ERK pathway to influence basal VSN development.

Also different from findings in the main olfactory epithelium, our study revealed that non-neuronal cell types, including horizontal basal cells and sustentacular cells, were affected by *Cxcr4* cKO. Specifically, we observed a significant increase in sustentacular cell numbers and alterations in proliferative status, as indicated by loss of MCM2 expression in *Cxcr4* cKO mice. Bromodeoxyuridine (BrdU)-labeling experiments of the VNO have shown that sustentacular cells might originate from the marginal zone, as do neuronal cells (Barber and Raisman, 1978). In context with our findings, this suggests a direct role of CXCR4 signaling in differentiation of yet unknown progenitor cells into sustentacular cells. Recent lineage tracing experiments using KRT5 to drive tdTomato expression demonstrated that horizontal basal cells originate from basal cells of the non-sensory epithelium within the marginal zone and migrate along the basal lamina, starting at birth until full coverage of the VNO with horizontal basal cells at post-natal day (P)60 (LeFever et al., 2024). CXCR4 staining was absent in basal cells of the non-sensory epithelium and horizontal basal cells. However, indirect effects on horizontal basal cell differentiation and migration due to changes in the microenvironment of the stem cell niche, possibly induced by an increase in globose basal cell abundance within the marginal zone, might explain the observed effects.

Re-exploration of a scRNAseq dataset by pseudotime analysis has been a useful approach to determine the timing of expression during differentiation of olfactory neurons and to identify transcription factors potentially contributing to receptor expression (Hussainy et al., 2022). Here, we go one step further and reanalyze available scRNAseq data using CellOracle, a combinational approach of pseudotime analysis together with perturbation analysis (Kamimoto et al., 2023). This computational knockout analysis for neuronal lineage-specific transcription factors in the VNO revealed 19 promising targets putatively affecting neurogenesis of the basal lineage of VSNs. Additional *in vivo* experiments are needed to confirm our predictions by determination of the VNO phenotypic changes in mice. Our results are a promising starting point and an advancement in deciphering the underlying gene regulatory network within the vomeronasal neuronal lineage.

In conclusion, our study reveals that CXCR4 signaling is crucial for regulating neurogenesis and for development of non-neuronal cell types in the VNO. The cKO of *Cxcr4* in globose basal cells leads to a reduction in DCX-expressing immediate neuronal precursors and an increase in OMP-expressing mature VSNs. Additionally, CXCR4 impacts the proliferation and differentiation of sustentacular cells and horizontal basal cells, suggesting it has a broad role in maintaining the VNO cellular environment. These findings highlight the importance of CXCR4 in ensuring proper sensory function and neuronal homeostasis in mice. Along with computational identification of transcription factors putatively involved in lineage-specific differentiation, our findings contribute to a deeper understanding of olfactory neurogenesis and potentially impact future research on function and regeneration of olfactory sensory organs.

## MATERIALS AND METHODS

### Animal breeding and treatment

Animal experiments adhered to the guidelines of the EC directive 86/609/European Economic Community for animal research and were approved by the local government (Thüringer Landesamt für Lebensmittelsicherheit und Verbraucherschutz). Mice were housed in a facility with a 12-h-light–12-h-dark cycle and provided *ad libitum* access to food and water. Initially, WT mice of C57BL6/6J background were obtained from Charles River Laboratories (Sulzfeld, GER). Transgenic *Htr3a*-Cre*;Cxcr4*^LoxP/LoxP^ mice were provided by Ralf Stumm and Dagmar Schütz (Jena, Germany). The *Htr3a*-Cre mouse strain (RRID:MMRRC_037089-UCD) expresses Cre recombinase driven by the serotonine receptor subunit 5-hydroxytryptamine receptor 3A (*5Htr3a*) promoter and was initially obtained from the Mutant Mouse Resource and Research Center (MMRRC) at University of California, Davis. *Cxcr4*^LoxP/LoxP^ mice initially originate from Dan Littman (Nie et al., 2004). *Cxcr4-Cre^ER^;R26^CAG-LSL-tdT^* mice were utilized for lineage tracing of *Cxcr4*-expressing cells within the VNO (Werner et al., 2020). Expression of the red fluorescence protein tandem Tomato (tdTomato) is induced by intraperitoneal injection with 50 mg/kg tamoxifen (Merck Millipore) at P21 and P28.

### Immunofluorescence and tissue preparation

For all experiments, mice of both sexes aged 8 weeks were euthanized with isoflurane and decapitated. The VNOs were the extracted and fixed in 4% paraformaldehyde (PFA; Carl Roth, Karlsruhe, Germany) for 24 h at 4°C. Subsequently, the fixed VNOs were cryopreserved in 30% sucrose and frozen in 2-methylbutane (Carl Roth) for immunofluorescence analysis. Tissues were embedded in tissue-freezing medium (Leica Microsystems, Wetzlar, Germany) on a specimen disk and sectioned coronally at a thickness of 18 µm. Immunostaining was conducted following established protocols, with details of primary and secondary antibodies provided in Tables S1 and S2; nuclear staining was achieved using Hoechst 33342 dye (Thermo Fisher Scientific, Waltham, MA, USA). Imaging was performed using either a confocal laser scanning microscope with TCS SPE system (Leica DM2500, Leica Microsystems) or Zeiss LSM900 with Airy-Scan technology (Carl Zeiss Microscopy GmbH, Oberkochen, Germany). For comparison of staining intensities or cell numbers, tissues were collected from mice kept in the same housing conditions, sections were stained in parallel and images taken for quantification were taken with identical settings of the microscope. Quantitative analysis involved studying comparable regions from three to five mice from different litters per group. Measurements of areas, thickness and cell counts were performed on digital pictures using ImageJ or ZEN 3.0 software. For this, the tissue was divided into six sections, each measured at a 30° angle as described (Naik et al., 2020) (Fig. S7A). For cell counting, we determined the background staining intensity in cells that did not express the protein of interest based on visual inspection and then counted cells which had intensity values that are a minimum of five times the background value as positive, cells were marked manually and counted using ImageJ (Fig. S7B). Cell counting was performed by at least two independent researchers that were aware of the experimental conditions. Intensity measurements were conducted using Fiji software, calculating integrated density values within regions of interest covering the relevant areas. Integrated density values were collected from six different areas per stained cryosection, placed in different areas of the epithelium (Fig. S7A) and one for background reference. Four to six VNE sections were quantified for each series and averaged. Data from each genotype were grouped and used for statistical analysis, a minimum of three different mice were analyzed per genotype. Intensities were determined relative to the areas analyzed and the intensity of the background reference. Image post-processing utilized software such as LAS X, ZEN 3.0 and ImageJ.

### Quantitative real-time PCR

Total RNA was extracted from VNO samples of 8-week-old WT and *Htr3a*-Cre*;Cxcr4*^LoxP/LoxP^ mice using the Purelink RNA Mini Kit (Thermo Fisher Scientific), cDNA was synthesized using the High Capacity cDNA Kit (Thermo Fisher Scientific). Quantitative real-time PCR (qPCR) was performed on a Quant Studio^®^ 3 Real Time PCR Cycler (Thermo Fisher Scientific) with predesigned Quantitect primers (Qiagen, Hilden, Germany) for *Dcx* (QT02521155), *Omp* (QT00257544), *Tfap2e* (QT00169358), *Gnao1* (QT00171108), *Gnai2* (QT00140469), *Neurog1* (QT00295064), *Neurod1* (QT00251265), *Ascl1* (QT00266861, *Pcdh7* (QT01052366) along with Power Up SYBR^®^ Green Master Mix (Thermo Fisher Scientific). Each genotype was represented by three to six animals, and three independent technical replicates were conducted for each cDNA per animal. The qPCR conditions comprised an initial denaturation step at 95.0°C for 2 min, followed by 44 cycles of denaturation at 95°C for 15 s, annealing and extension at 55°C for 1 min, and a final extension at 60°C for 1 min. mRNA expression levels were quantified using the ΔΔCT method and presented as 2^−ΔΔCT^ values relative to *Gapdh* expression (Schmittgen and Livak, 2008).

### *In situ* hybridization

Fluorescent *in situ* hybridization was performed on 20 µm cryosections of olfactory epithelium from adult (8 weeks old) mice, fresh frozen in 2-methylbutane. Sections were fixed in ice-cold 4% PFA for 1 h, and permeabilized in 0.4% Triton X-100 triethanolamine buffer (pH 8) and acetic anhydride. For probe construction, mouse *Atf5* cDNA was amplified from murine olfactory epithelium using PCR and cloned into pCDNA3 (amplified region, 299–1151 nt; 853 bp; gene ID, 107503; NM030693). DIG probe synthesis was undertaken as previously described (Senf et al., 2021). For hybridization, sections were incubated with 2 µg/ml *Atf5* riboprobe in hybridization buffer for 20 h at 60°C in a humid chamber containing 50% formamide. Afterwands, sections were rinsed in 2× Tri-sodium-citrate-dihydrate (SSC), 1× SSC at room temperature, RNAse solution for 30 min at 42°C, followed by 0.2× SSC at room temperature and a hot wash in 0.2× SSC for for 1 h. Sections were transferred into maleic acid and incubated in blocking buffer for 1 h at room temperature. For 30 min sections were incubated in Anti-Digoxigenin-POD Fab fragments (1:500), followed by washing in maleic acid. For visualization, slides were treated with TSA™ Plus Cyanine 3 Amplification Kit for 15 min in the dark at room temperature, washed again in maleic acid and mounted with Fluoromount-G™. Images were performed using a confocal laser scanning microscope with TCS SPE system (Leica DM2500. Leica Microsystems, Wetzlar, GER).

### Single cell RNA dataset analysis

The previously published single-cell RNA sequencing (scRNAseq) dataset of P60 mice was obtained from the gene express omnibus database under accession number GSE190330 (Katreddi et al., 2022). Clusters were annotated based on gene expression as described previously (Katreddi et al., 2022): basal cells (*Trp63* and *Krt5*), stem cell progenitors (*Sox2* and *Ascl1*), neural precursors (*Neurog1* and *Neurod1*), immature neurons (*Gap43*), mature VSNs (*Omp*), sustentacular cells (*Fezf2* and *Cyp2a5*), olfactory ensheathing cells (*S100b*, *Plp1*, *Mpz* and *Sox10*), pericytes (*Pecam1*, *Eng* and *Sox17*), vascular smooth muscle cells (*Tagln* and *Acta2*), Vegfa+ cells (*Vegfa*), T cells (*Cd3d* and *Cd3e*), B cells (*Cd19* and *Cd79a*), macrophages (*C1qa* and *C1qb*), monocytes (*Chil3*, *Clec10a* and *Ccr2*). The dataset was processed and subsetted to delineate the immature neuronal lineage of the VNO following the methodology published (Katreddi et al., 2022), utilizing the R version 4.2.2 (https://www.r-project.org/) with the Seurat package 4.2 (Hao et al., 2021), marker genes for basal VSNs were *Gnao*, *Robo2* and V2-type vomeronasal receptors, markers for apical VSNs were *Gnai2*, *Nrp2* and V1-type vomeronasal receptors. Visualization of feature plots and dot plots was accomplished using built-in functions of Seurat.

### CellOracle computational gene perturbation analysis

Lineage tracing and gene perturbation analysis of the GSE190330 scRNAseq neuronal subset dataset were conducted using the Python library CellOracle (Kamimoto et al., 2023). CellOracle version 0.14.0 was installed within an Anaconda environment (Anaconda Software Distribution, Vers. 2.4.0) running Python 3.8 on an ARM MacBook Pro with MacOS Sonoma 14.0. Installation was performed using the gcc compiler and XCode command line tools, following the guidelines in the CellOracle documentation. The web-based Jupyter Notebook application was utilized for code execution. The neuronal subset data was exported as a matrix file using the R software package DropletUtils (Griffiths et al., 2018; Lun et al., 2019) and subsequently loaded into CellOracle. Clustering and UMAP projection were performed with parameters set to *n*_neighbors=10 and *n*_pcs=50, and resulting clusters were annotated similar to Seurat clustering. The cell identity GGGTATTAGGAATGTT-1 was set as the root cell for pseudotime calculation. Gene regulatory networks (GRNs) for all clusters were calculated using the prebuilt promoter base GRN obtained from the mouse reference genome in version mm10_gimmemotifsv5_fpr2 (van Heeringen and Veenstra, 2011). Processing followed the standard workflow according to the CellOracle documentation.

### Statistical analysis

Statistical analysis was performed using R Version 4.2.2 (R Core Team, 2018), with data presented as box plots. Normal distribution and variance homogeneity of the data were assessed. Statistical significance was determined at *P<0.05 and analyzed using either a two-tailed unpaired Student's *t*-test or a Welch test, as appropriate. Power values were between 81 and 99%.

## Supplementary Material



10.1242/joces.263451_sup1 Supplementary information
