# Supplementary material for: Stem cell expression of CXCR4 regulates tissue composition in the vomeronasal organ
Source: J Cell Sci. 2025 Jan 9;138(1):jcs263451. doi: 10.1242/jcs.263451 (PMC11828470; doi:10.1242/jcs.263451)
Supplement: Supplementary information [file joces-138-263451-s1.pdf]

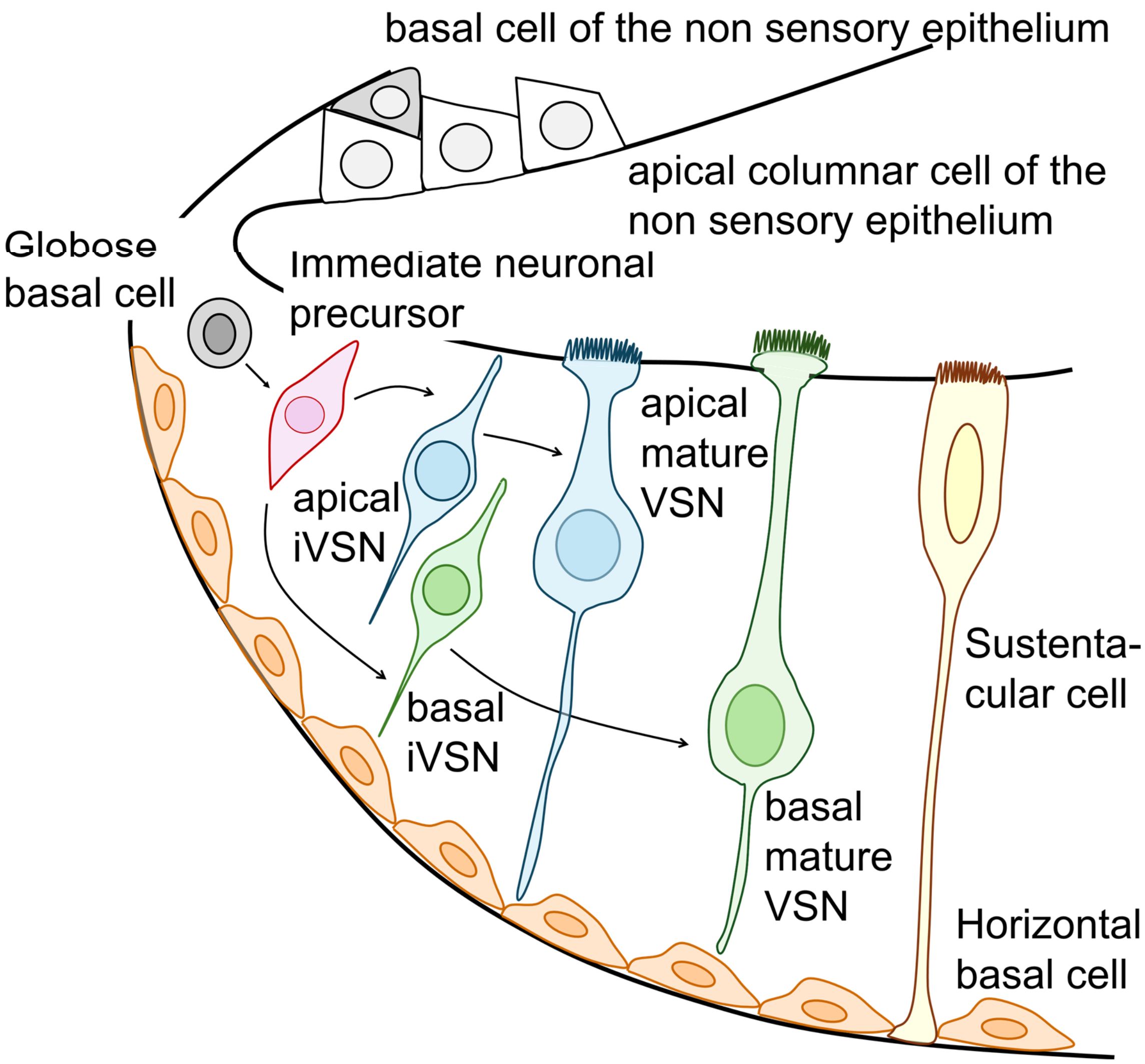

Fig. S1. Schematic illustration showing common cell types of the VNO with focus on neurogenesis in the marginal zone of the sensory epithelium, facing apically the non-sensory epithelium.

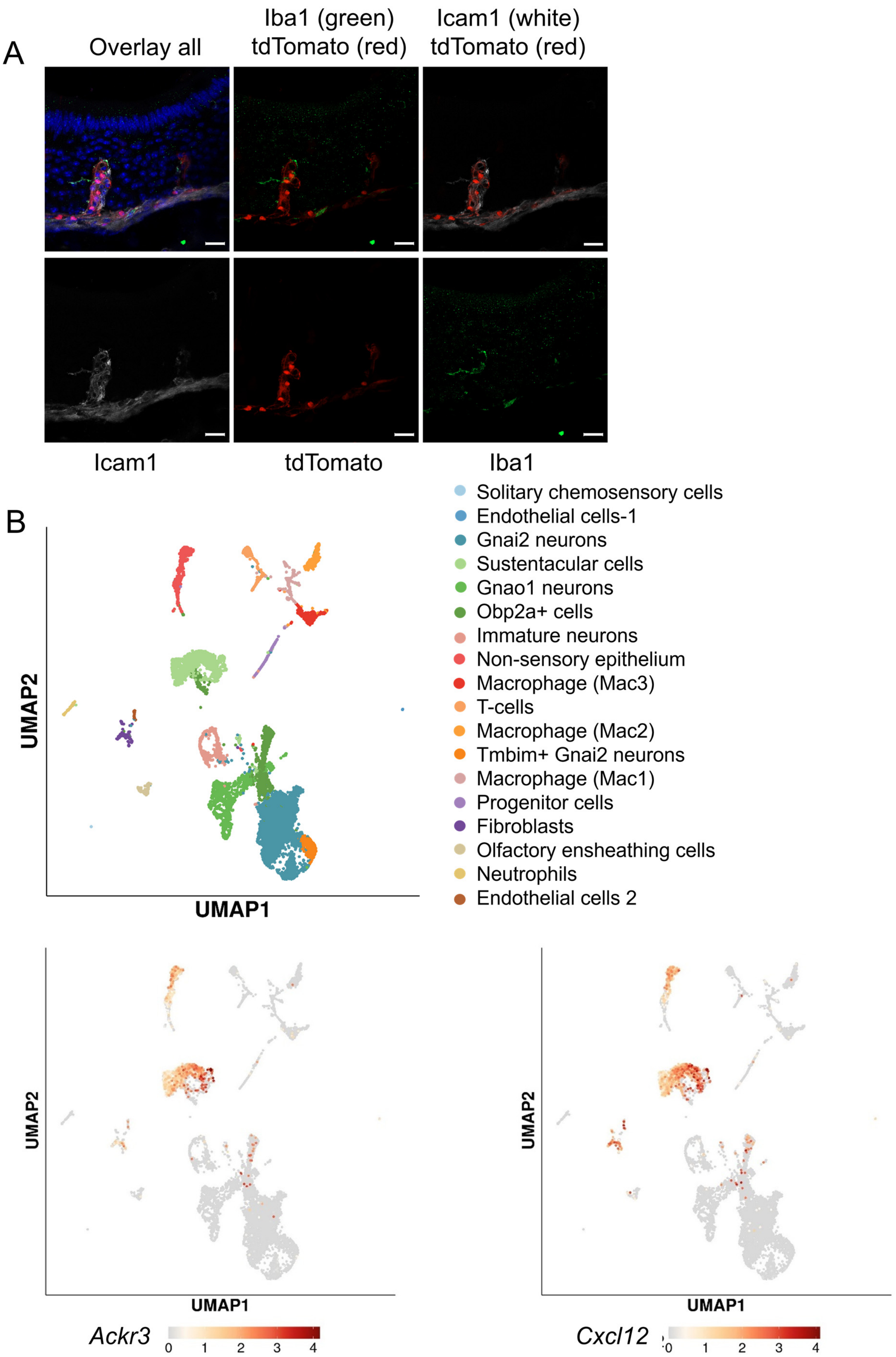

Journal of Cell Science • Supplementary information

Fig. S2. *Cxcr4*, *Ackr3* and *Cxcl12* are expressed in different structures of the VNO.

(**A**) Immunofluorescence staining of *Cxcr4*-Cre mice with tdTomato (red), the macrophage marker IBA1 (green) and ICAM1 (white) as marker for blood vessel. Single channels for overlay shown Fig. 1D. in WT and *Cxcr4* cKO mice. Scale bars 20 µm. (**B)** Analysis of the publicly available web page accessible single-cell RNA explorer for the VNO, discriminating also in cells of the non-sensory epithelium, shows co-expression of *Ackr3* and *Cxcl12* in cells of the non-sensory epithelium and sustentacular cells (www.scvnoexplorer.com) (Gvs et al., 2024).

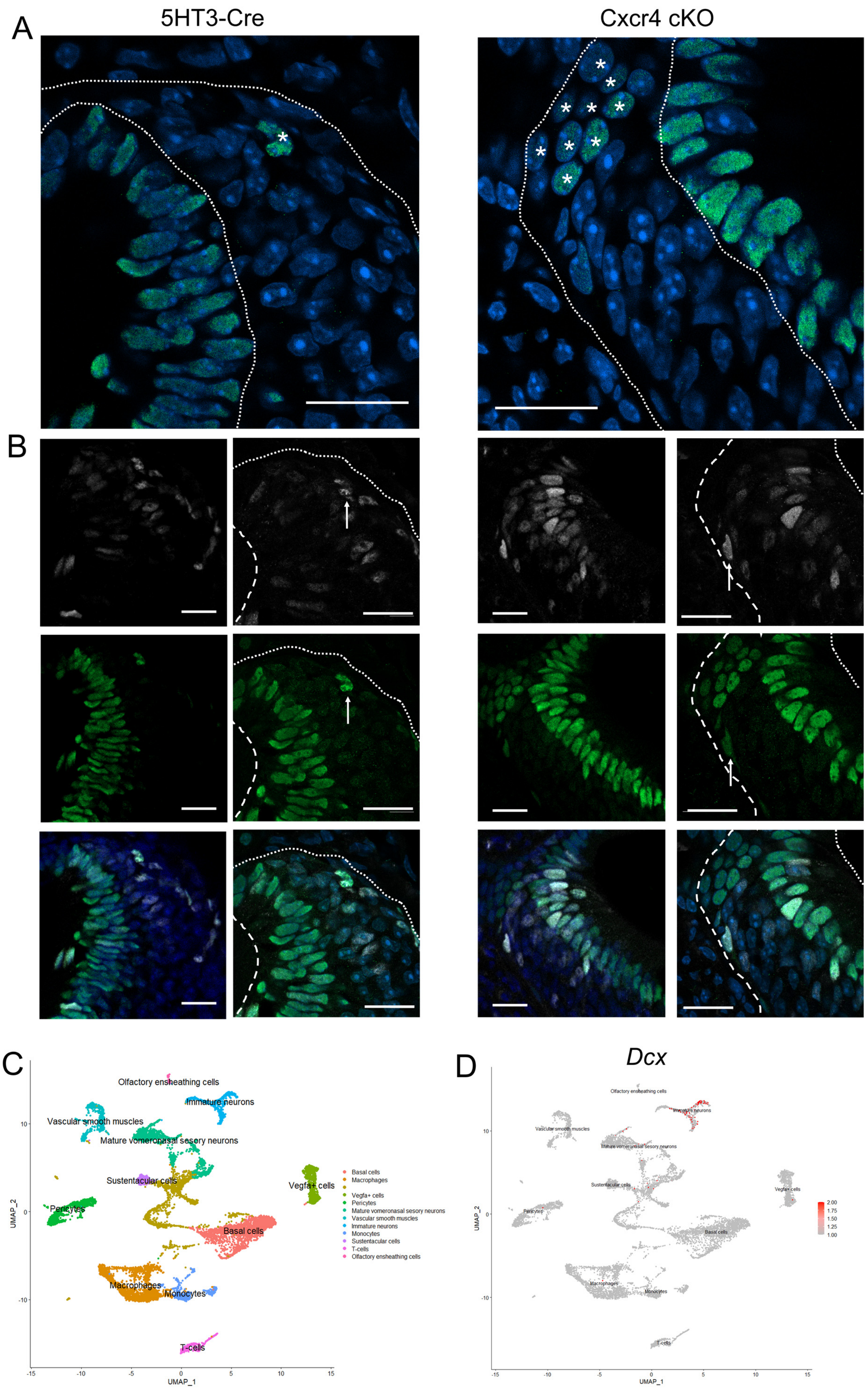
A
5HT3-Cre
Cxcr4 cKO
B
C
Olfactory ensheathing cells
Immature neurons
Vascular smooth muscles
Mature vomeronasal sesory neurons
Sustentacular cells
Vegfa+ cells
Pericytes
Basal cells
Macrophages
Monocytes
T-cells
Basal cells
Macrophages
Vegfa+ cells
Pericytes
Mature vomeronasal sesory neurons
Vascular smooth muscles
Immature neurons
Monocytes
Sustentacular cells
T-cells
Olfactory ensheathing cells
UMAP_1
UMAP_2
D
Dcx
2.00
1.75
1.50
1.25
1.00

Fig. S3. Co-labeling of SOX2 and MCM2 in the marginal zone

(A) Labeling of nuclei (Hoechst) allows discrimination between elongated nuclei of sustentacular cells (above the dotted line) and nuclei of other cells of the marginal zone (area between both dotted lines). SOX2-positive nuclei (green) are labeled with white asterisks. (**B**) Immunofluorescence staining of SOX2 (green), MCM2 (white) in WT and *Cxcr4* cKO mice, nuclei (blue). Nuclei that are positive for both markers are labeled by white arrows. (**C**) Reproduction of complete UMAP reduction plot of scRNAseq dataset GSE190330 (Katreddi et al., 2022). **(D)** Visualization of cells expressing *Dcx* shows localization in all mature VSNs. Fine dotted lines represents the basal limitation, dashed lines the apical limitation of the VNO. Scale bars 20 µm.

Journal of Cell Science • Supplementary information

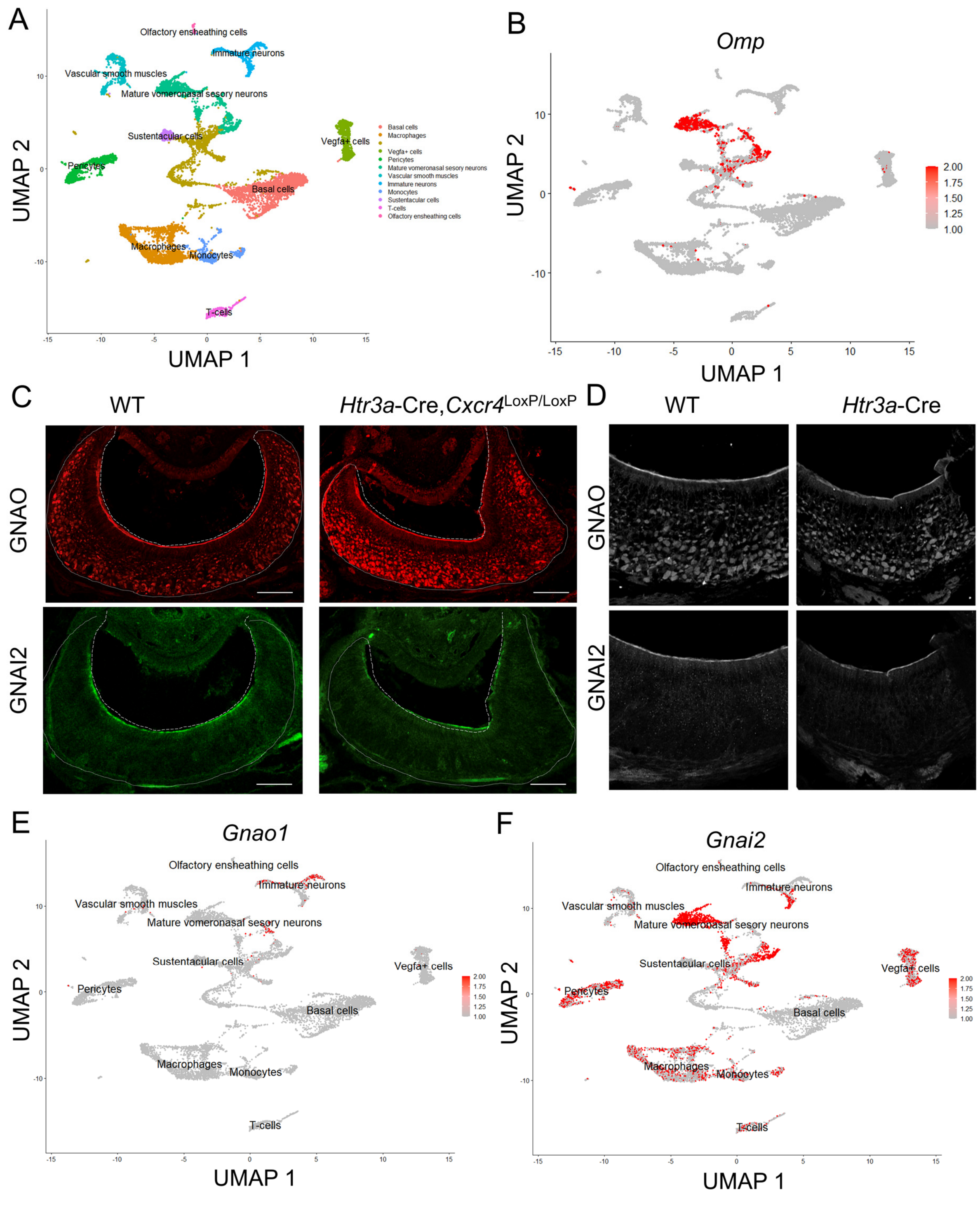

Fig. S4. *Cxcr4* cKO leads to increase in basal neuronal population size.
(A) Reproduction of complete UMAP reduction plot of scRNAseq dataset GSE190330 (Katreddi et al., 2022). **(B)** Visualization of cells expressing *Omp* shows localization in all mature VSNs. (**C**) Immunofluorescence staining of GNAO (red, upper panel) and GNAI2 (green, lower panel) in WT and *Cxcr4* cKO mice. **(D)** Immunofluorescence staining of GNAO (upper panel) and GNAI2 (lower panel) in WT and

Journal of Cell Science • Supplementary information

Htr3a-Cre mice (driver line for *Cxcr4* cKO*)* showing no difference. **(E)** Visualization of cells expressing *Gnao* within the complete VNO dataset shows localization in basal immature and mature basal VSNs. **(F)** Visualization of cells expressing *Gnai2* within the complete VNO dataset shows localization in multiple cell types (basal immature and mature apical VSNs, pericytes, macrophages, monocytes, Vegfa+ cells, sustentacular cells). Fine dotted lines represent the basal limitation, dashed lines the apical limitation of the VNO. Scale bars **(C)** 100 µm, **(D)** 20 µm.

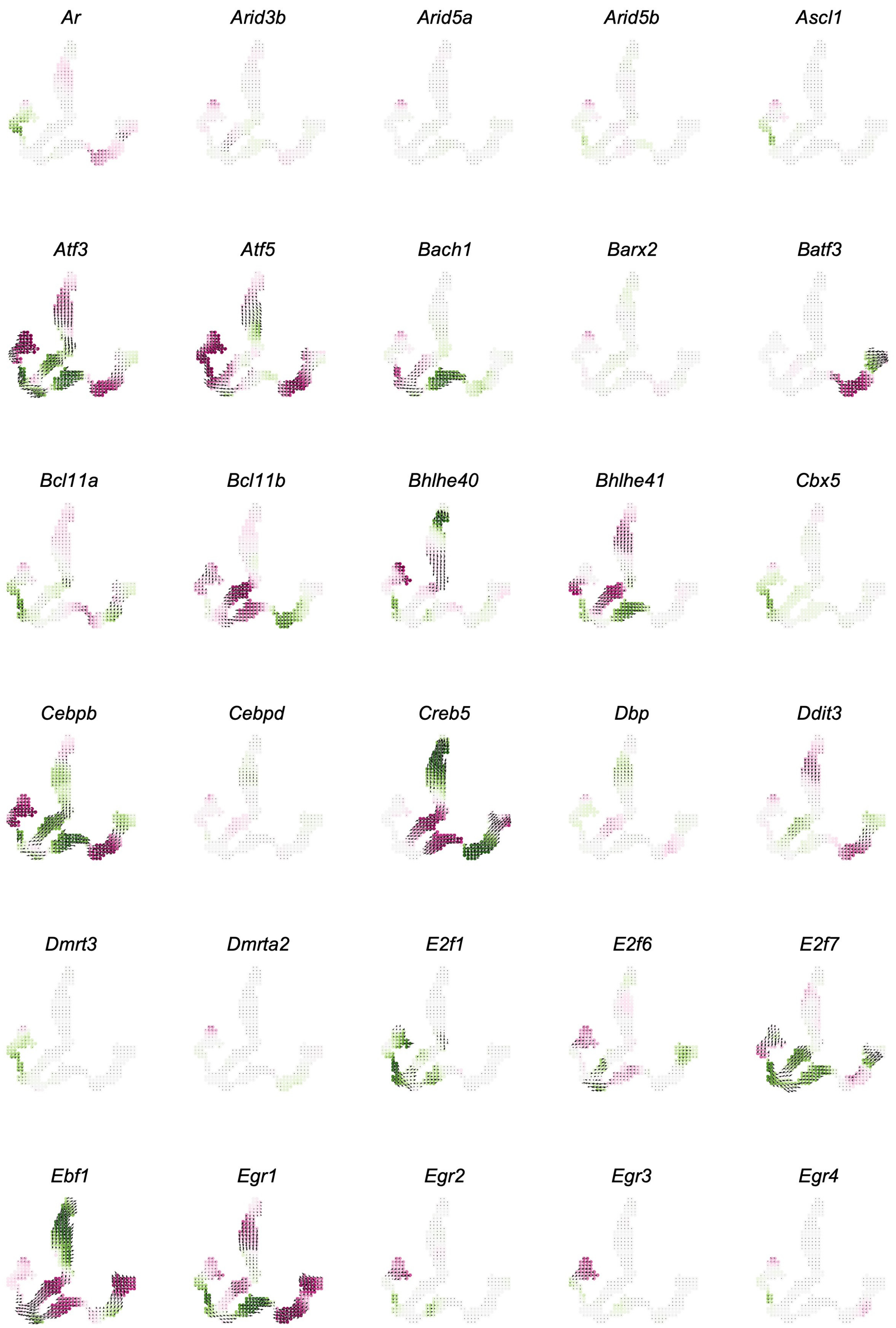
Ar
Arid3b
Arid5a
Arid5b
Ascl1
Atf3
Atf5
Bach1
Barx2
Batf3
Bcl11a
Bcl11b
Bhlhe40
Bhlhe41
Cbx5
Cebpb
Cebpd
Creb5
Dbp
Ddit3
Dmrt3
Dmrta2
E2f1
E2f6
E2f7
Ebf1
Egr1
Egr2
Egr3
Egr4

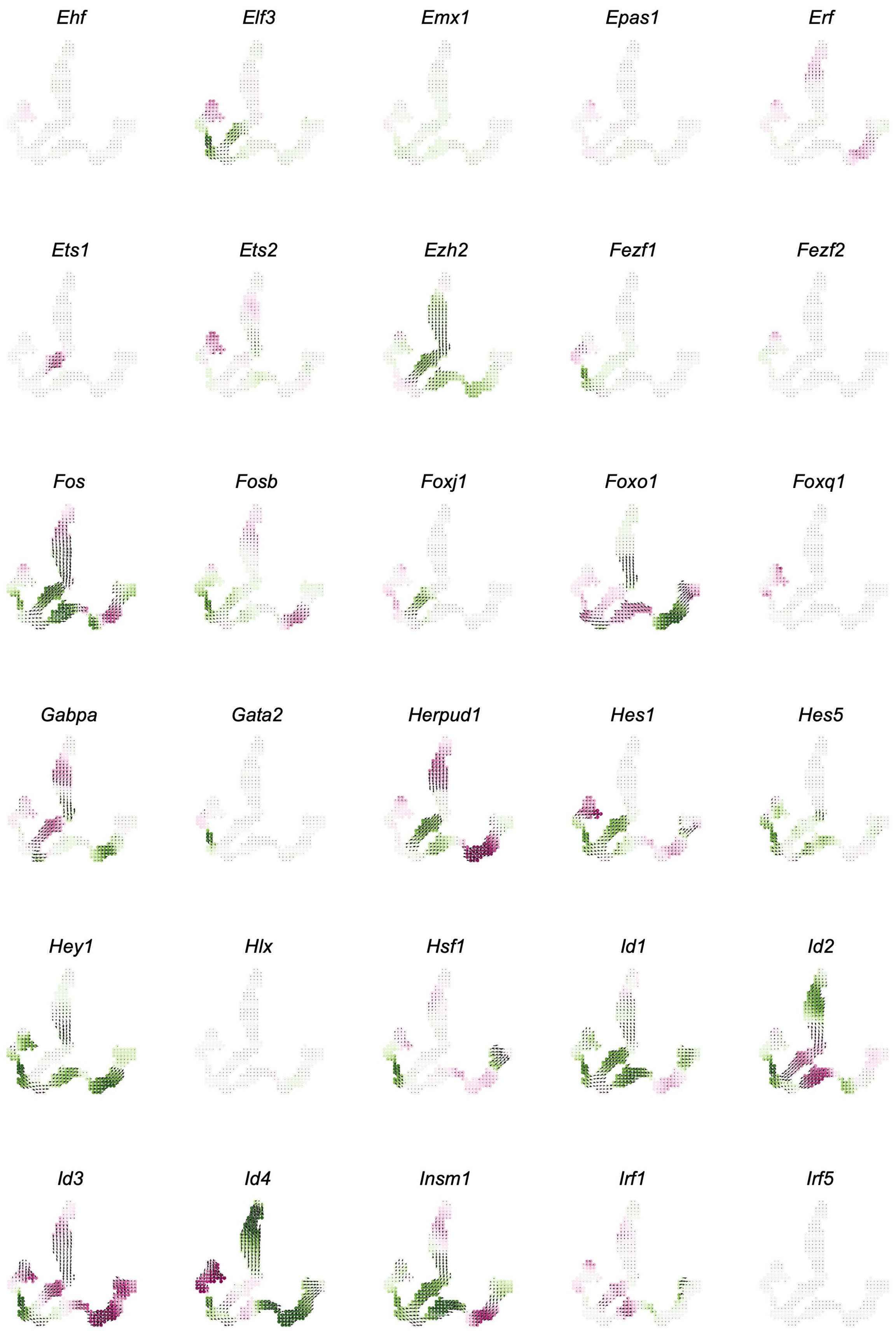
Ehf
Elf3
Emx1
Epas1
Erf
Ets1
Ets2
Ezh2
Fezf1
Fezf2
Fos
Fosb
Foxj1
Foxo1
Foxq1
Gabpa
Gata2
Herpud1
Hes1
Hes5
Hey1
Hlx
Hsf1
Id1
Id2
Id3
Id4
Insm1
Irf1
Irf5

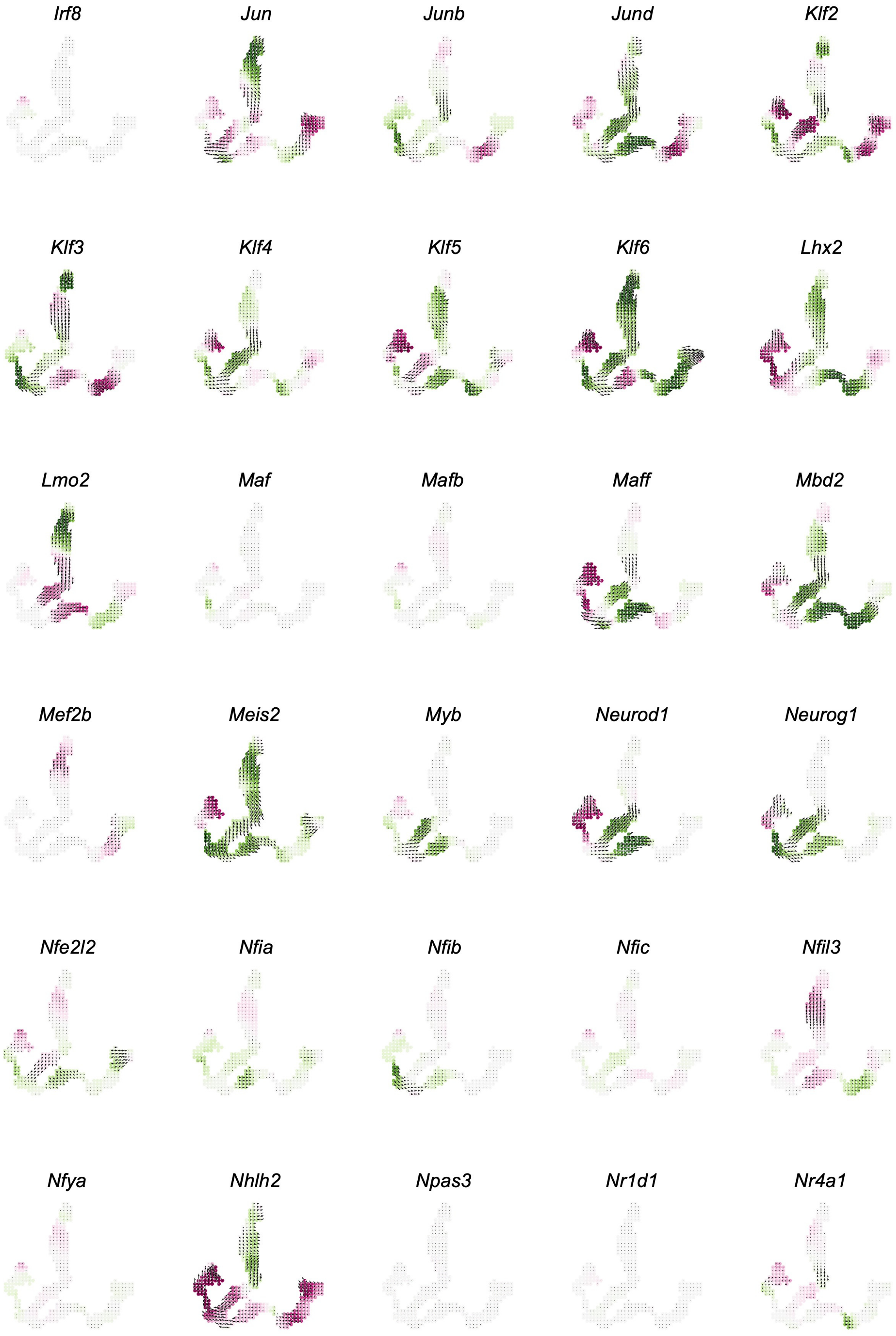

Journal of Cell Science • Supplementary information

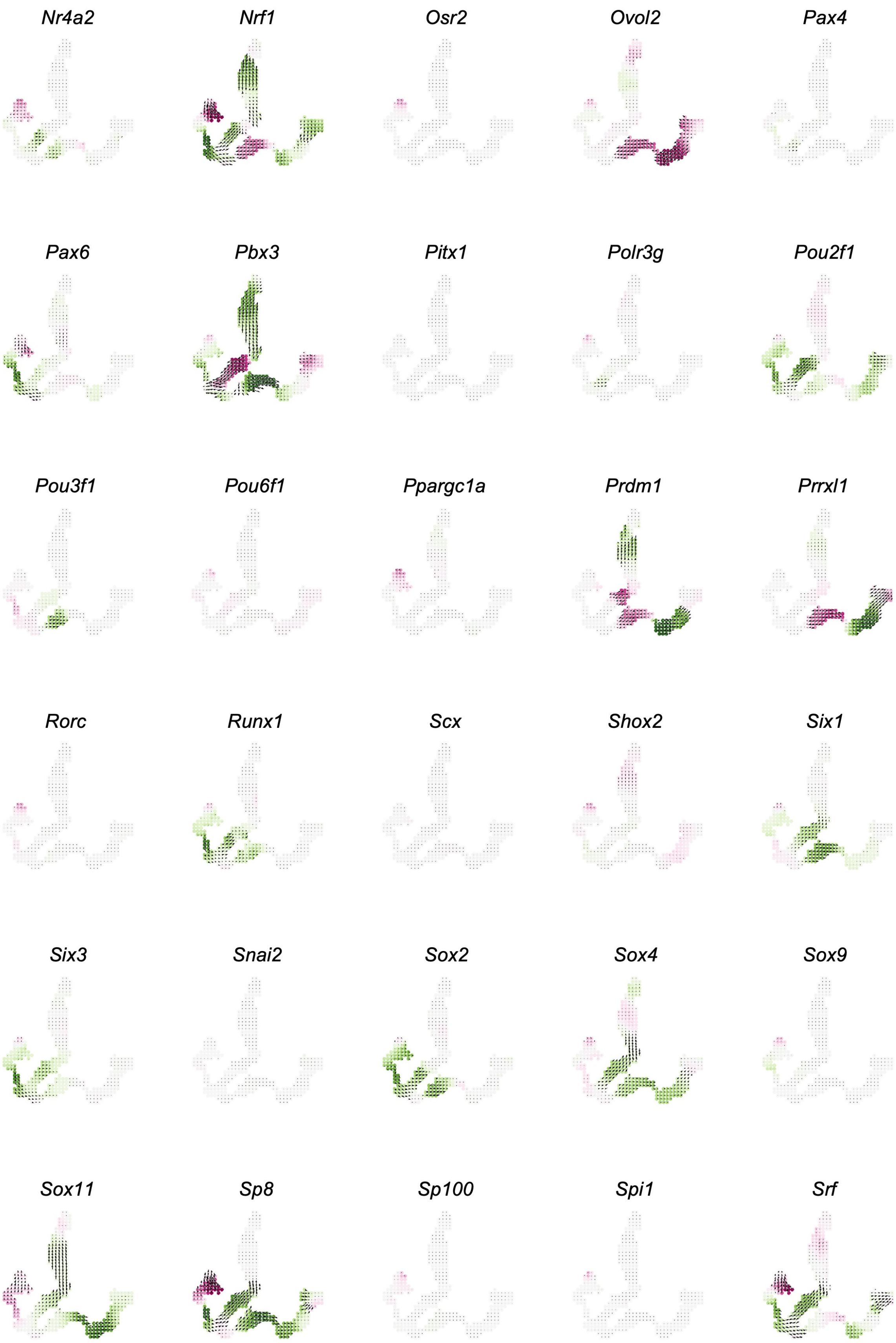
Nr4a2
Nrf1
Osr2
Ovol2
Pax4
Pax6
Pbx3
Pitx1
Polr3g
Pou2f1
Pou3f1
Pou6f1
Ppargc1a
Prdm1
Prrxl1
Rorc
Runx1
Scx
Shox2
Six1
Six3
Snai2
Sox2
Sox4
Sox9
Sox11
Sp8
Sp100
Spi1
Srf

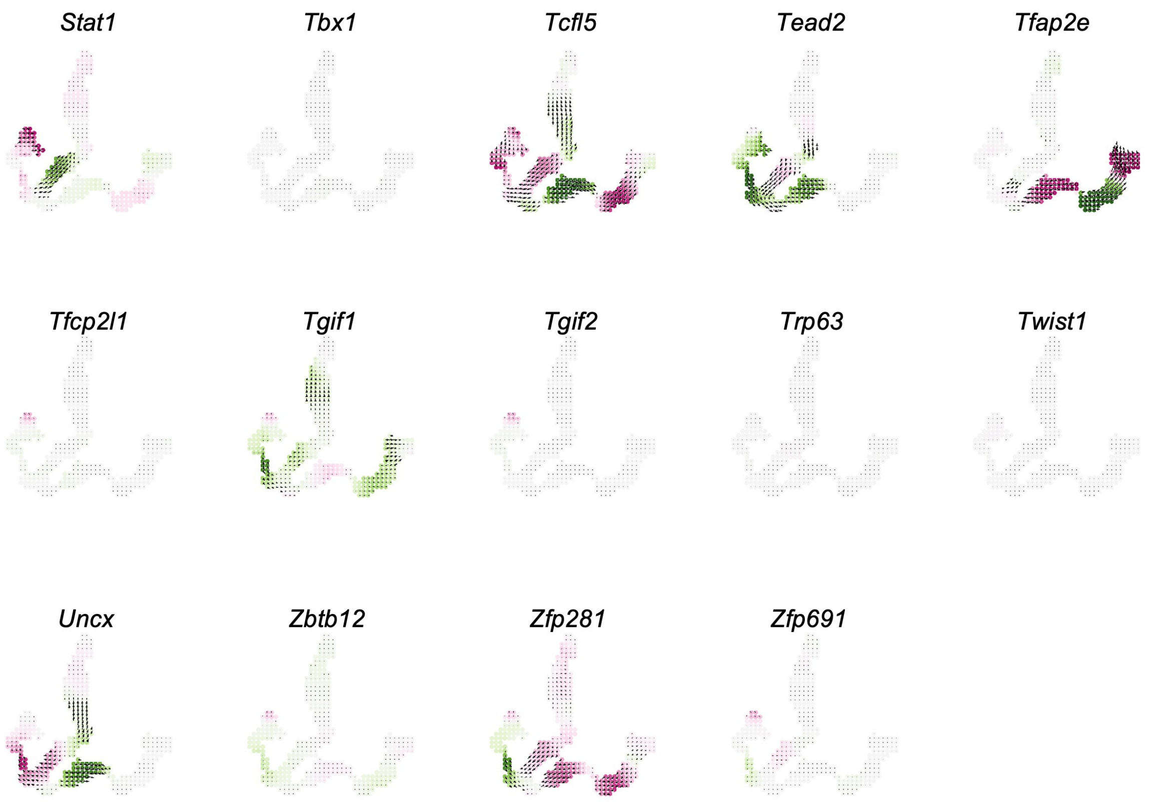

Fig. S5. CellOracle in silico perturbation plots.

Knockout perturbation analysis for every modifiable transcription factor identified by CellOracle for the neuronal subset of the VNO. *Ascl1*, *Atf5*, *Bcl11b*, *Fezf2*, *Neurog1*, *Pax6*, and *Six1* revealed a different outcome to published results on developmental neurogenesis (Cau et al., 2002; Eckler et al., 2011; Enomoto et al., 2011; Grindley et al., 1995; Ikeda et al., 2010; Murray et al., 2003; Nakano et al., 2016). Vector maps show transitions in cell identity, color codes represent the directionality of the perturbation vector. Positive perturbation scores (green) indicate that knock-out of these transcription factors promotes neuronal differentiation, negative perturbation scores (purple) show reduced differentiation. Fig.S3 encompasses 5 pages.

Journal of Cell Science • Supplementary information

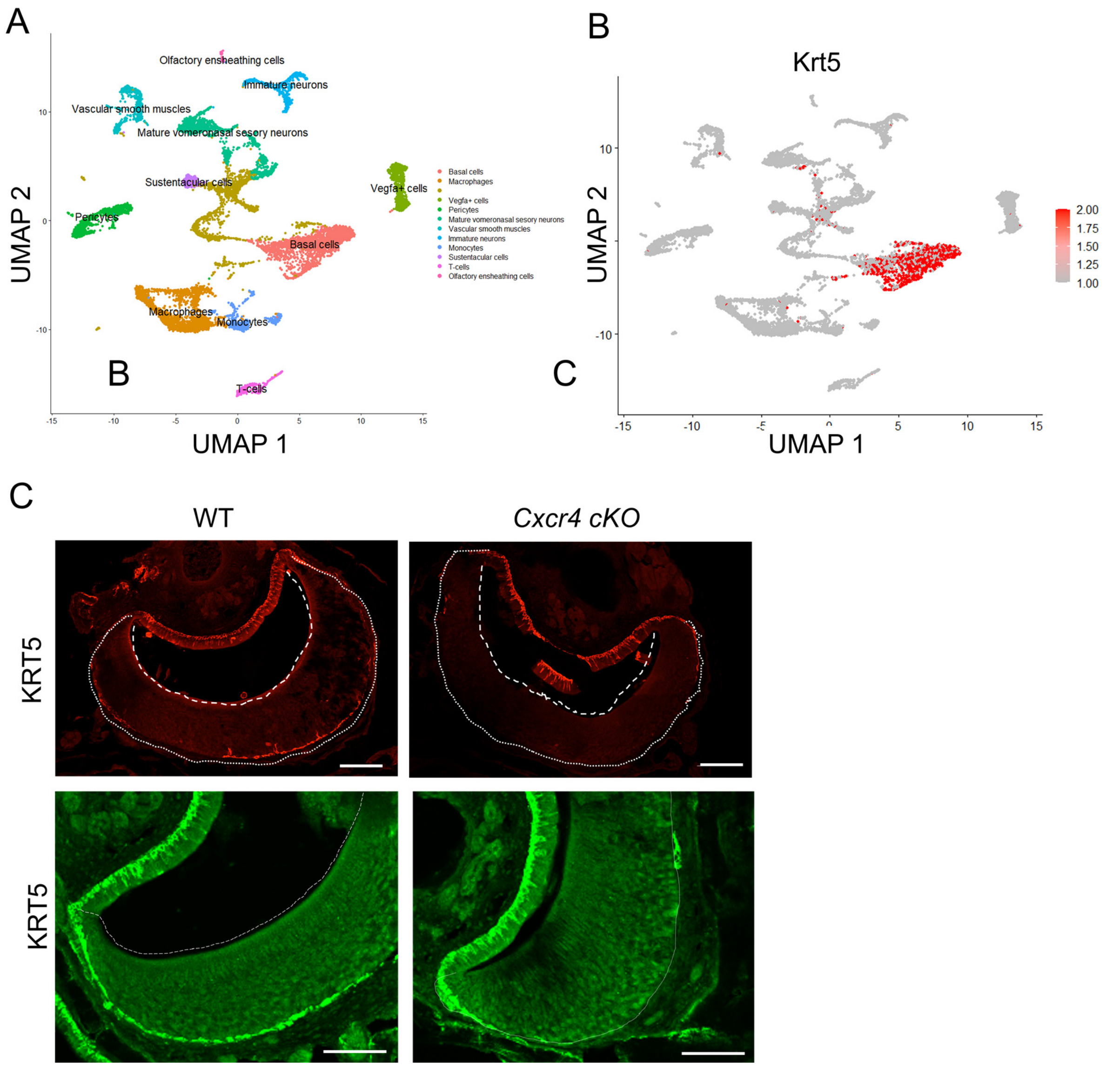

Fig.S6. *Cxcr4* cKO leads to decrease in horizontal basal cells

(A) Reproduction of complete UMAP reduction plot of scRNAseq dataset GSE190330 (Katreddi et al., 2022). **(B)** Visualization of cells expressing *Krt5* shows localization in horizontal basal cells. (**C**) Immunofluorescence staining of KRT5 (red) in WT and *Cxcr4* cKO mice. (**D**) Immunofluorescence staining of KRT5 (red) in WT and *Cxcr4* cKO mice. Fine dotted lines represent the basal limitation, dashed lines the apical limitation of the VNO. Scale bars 50 µm.

Journal of Cell Science • Supplementary information

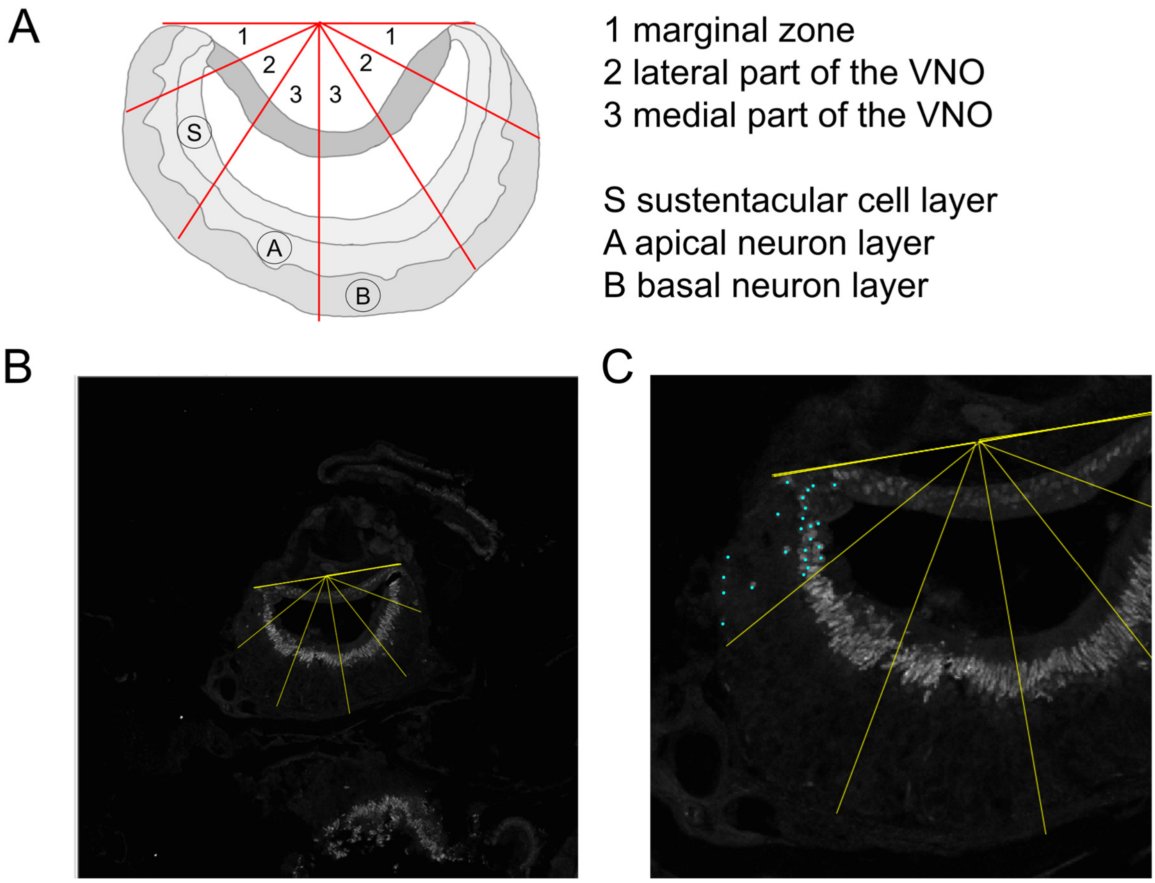

Fig. S7. Quantification of immunofluorescence images.

Cartoon illustrating AP-2εCre recombination in basal VSNs. Lines and numbers indicate the seven different sectors of the VNO, where sectors 1 and 7 are the marginal regions, which is where neurogenesis occurs, and 2-6 are medial.

Journal of Cell Science • Supplementary information

Table S1. Primary antibodies

antibody target	host species	Dilution	company (order number)	Antibody characterization
CXCR4 (2B11, N-term)	rat	1:500	Affymetrix (14-9991-82)	Recognizes the amino terminus of mouse CXCR4, labeling absent in CXCR4 KO animals (Senf et al., 2021)
RFP	goat	1:200	Sicgen (AB8181)	Goat polyclonal antibody to tdTomato (red fluorescent protein), detects tdTomato in brain sections by IHC and WB, does not cross-react with GFP (manufacturer information), used in 48 publications (according to manufacturer).
AP2ε	goat	1:100	R&D Systems (AF5060)	Polyclonal antibody that detects human AP-2 epsilon in direct ELISAs and Western blots. In direct ELISAs and Western blots, less than 5% cross-reactivity with recombinant human (rh) AP-2 alpha, rhAP-2 beta, rhAP-2δ, and rhAP-2 gamma is observed. Published use in immunofluorescence experiments of the vomeronasal organ (Katreddi et al., 2022; Lin et al., 2022).
SOX2	goat	1:100	R&D Systems (AF2018)	Polyclonal antibody against human, mouse, rat SOX2. Used in 351 publications according to manufacturer homepage.
MCM2 (BM-28)	mouse	1:200	BD Biosciences (610700)	Raised against human BM28 aa. 725-888, reactive to mouse protein.
DCX	guinea pig	1:100	Synaptic Systems (326 004)	Polyclonal antibody against recombinant protein corresponding to AA 271 to 366 from mouse Doublecortin. Shown to work in central nervous system immunofluorescence experiments (Gallardo-Caballero et al., 2023).
OMP	goat	1:500	Wako (544-10001)	Multiple immunizations of rodent olfactory marker protein, widely used to stain olfactory neurons, recapitulates the canonical staining pattern for OMP described for the goat anti-OMP originally distributed by Frank Margolis (Keller and Margolis, 1976), extensively characterized previously (Rodriguez-Gil and Greer, 2008).
Gαo	rabbit	1:50	Santa Cruz (sc-387)	Monoclonal antibody against highly divergent domain of Gαo of rat origin. Used in 46 publications (according to manufacturer).

Journal of Cell Science • Supplementary information

Gαi2	rat	1:200	Santa Cruz (sc-13534)	Polyclonal antibody. Used in 75 publications (according to manufacturer). Used in several publications for VNO immunostaining, e.g. (Kondoh et al., 2022; Liu et al., 2019).
KRT5	rabbit	1:500	Convance (PRB-160P)	Raised against a peptide sequence derived from the C-terminus of the mouse keratin 5 protein, used to stain horizontal basal cells in the olfactory epithelium, e.g. (Chen et al., 2019).
CAR2	Rabbit	1:100	Abcam (Ab191343)	Polyclonal antibody against native full length protein corresponding to Human CAR2. Used in 17 publications (according to manufacturer).
OVOL2	rabbit	1:100	Thermofisher Scientific (PA5-115700)	Polyclonal antibody against synthesized peptide derived from human OVOL2 corresponding to amino acid residues V200-L250.
NCAM2	goat	1:50	R&D Systems (AF-778-SP)	Goat Polyclonal Antibody against recombinant mouse OCAM Leu20-Gly700 that has been used in 8 publications according to the manufacturer. Less than 1% cross-reactivity with recombinant mouse NCAM1.

Table S2. Secondary antibodies.

secondary antibody	company (order number)
Alexa Fluor® 488 donkey anti-goat	Thermo Fisher Scientific (A-11055)
Alexa Fluor® 488 donkey anti-mouse	Thermo Fisher Scientific (A-21202)
Alexa Fluor® 488 donkey anti-rabbit	Thermo Fisher Scientific (A-21206)
Alexa Fluor® 488 donkey anti-rat	Thermo Fisher Scientific (A-21208)
Alexa Fluor® 568 donkey anti-goat	Thermo Fisher Scientific (A-11057)
Alexa Fluor® 568 donkey anti-guinea pig	Thermo Fisher Scientific (A-10037)
Alexa Fluor® 568 donkey anti-rabbit	Thermo Fisher Scientific (A-10042)
Alexa Fluor® 647 donkey anti-rabbit	Thermo Fisher Scientific (A-31573)
Alexa Fluor® 647 donkey anti-rat	Jackson Immuno Research (712-605-153)

References

Cau, E., Casarosa, S. and Guillemot, F. (2002). Mash1 and Ngn1 control distinct steps of determination and differentiation in the olfactory sensory neuron lineage. *Development* **129**, 1871-80.

Chen, M., Reed, R. R. and Lane, A. P. (2019). Chronic Inflammation Directs an Olfactory Stem Cell Functional Switch from Neuroregeneration to Immune Defense. *Cell Stem Cell* **25**, 501-513 e5.

Journal of Cell Science • Supplementary information

Eckler, M. J., McKenna, W. L., Taghvaei, S., McConnell, S. K. and Chen, B. (2011). Fezf1 and Fezf2 are required for olfactory development and sensory neuron identity. *J Comp Neurol* **519**, 1829-46.

Enomoto, T., Ohmoto, M., Iwata, T., Uno, A., Saitou, M., Yamaguchi, T., Kominami, R., Matsumoto, I. and Hirota, J. (2011). Bcl11b/Ctip2 controls the differentiation of vomeronasal sensory neurons in mice. *J Neurosci* **31**, 10159-73.

Gallardo-Caballero, M., Rodriguez-Moreno, C. B., Alvarez-Mendez, L., Terreros-Roncal, J., Flor-Garcia, M., Moreno-Jimenez, E. P., Rabano, A. and Llorens-Martin, M. (2023). Prolonged fixation and post-mortem delay impede the study of adult neurogenesis in mice. *Commun Biol* **6**, 978.

Grindley, J. C., Davidson, D. R. and Hill, R. E. (1995). The role of Pax-6 in eye and nasal development. *Development* **121**, 1433-42.

Gvs, D., Terasaki, M. and Dani, A. (2024). Single cell transcriptomics of vomeronasal neuroepithelium reveals a differential endoplasmic reticulum environment amongst neuronal subtypes: eLife Sciences Publications, Ltd.

Ikeda, K., Kageyama, R., Suzuki, Y. and Kawakami, K. (2010). Six1 is indispensable for production of functional progenitor cells during olfactory epithelial development. *Int J Dev Biol* **54**, 1453-64.

Katreddi, R. R., Taroc, E. Z. M., Hicks, S. M., Lin, J. M., Liu, S., Xiang, M. and Forni, P. E. (2022). Notch signaling determines cell-fate specification of the two main types of vomeronasal neurons of rodents. *Development* **149**.

Keller, A. and Margolis, F. L. (1976). Isolation and characterization of rat olfactory marker protein. *J Biol Chem* **251**, 6232-7.

Kondoh, D., Kawai, Y. K., Watanabe, K. and Muranishi, Y. (2022). Artiodactyl livestock species have a uniform vomeronasal system with a vomeronasal type 1 receptor (V1R) pathway. *Tissue Cell* **77**, 101863.

Lin, J. M., Mitchell, T. A., Rothstein, M., Pehl, A., Taroc, E. Z. M., Katreddi, R. R., Parra, K. E., Zuloaga, D. G., Simoes-Costa, M. and Forni, P. E. (2022). Sociosexual behavior requires both activating and repressive roles of Tfap2e/AP-2ε in vomeronasal sensory neurons. *Elife* **11**.

Liu, Q., Zhang, Y., Wang, P., Guo, X., Wu, Y., Zhang, J. X. and Huang, L. (2019). Two Preputial Gland-Secreted Pheromones Evoke Sexually Dimorphic Neural Pathways in the Mouse Vomeronasal System. *Front Cell Neurosci* **13**, 455.

Murray, R. C., Navi, D., Fesenko, J., Lander, A. D. and Calof, A. L. (2003). Widespread defects in the primary olfactory pathway caused by loss of Mash1 function. *J Neurosci* **23**, 1769-80.

Nakano, H., Iida, Y., Suzuki, M., Aoki, M., Umemura, M., Takahashi, S. and Takahashi, Y. (2016). Activating transcription factor 5 (ATF5) is essential for the maturation and survival of mouse basal vomeronasal sensory neurons. *Cell Tissue Res* **363**, 621-33.

Rodriguez-Gil, D. J. and Greer, C. A. (2008). Wnt/Frizzled family members mediate olfactory sensory neuron axon extension. *J Comp Neurol* **511**, 301-17.

Senf, K., Karius, J., Stumm, R. and Neuhaus, E. M. (2021). Chemokine signaling is required for homeostatic and injury-induced neurogenesis in the olfactory epithelium. *Stem Cells* **39**, 617-635.
