# Supplementary material for: Stem cell expression of CXCR4 regulates tissue composition in the vomeronasal organ
Source: J Cell Sci. 2025 Jan 9;138(1):jcs263451. doi: 10.1242/jcs.263451 (PMC11828470; doi:10.1242/jcs.263451)
Supplement: Supplementary file 1 [file joces-138-263451_review_history.pdf]

Journal of Cell Science

Stem cell expressed CXCR4 regulates tissue composition in the vomeronasal organ

André Dietz, Katja Senf and Eva M. Neuhaus
DOI: 10.1242/jcs.263451

Editor: Caroline Hill

Review timeline

Original submission:	24 July 2024
Editorial decision:	29 August 2024
First revision received:	5 November 2024
Editorial decision:	25 November 2024
Second revision received:	27 November 2024
Accepted:	1 December 2024

Original submission

First decision letter

MS ID#: JOCES/2024/263451

MS TITLE: Expanding the field of view: A simple approach for interactive visualisation of electron microscopy data at continuous magnification.

AUTHORS: André Dietz; Katja Senf; Eva M. Neuhaus
ARTICLE TYPE: Research Article

We have now reached a decision on the above manuscript.

To see the reviewers' reports and a copy of this decision letter, please go to:

As you will see, the reviewers raise a number of substantial criticisms that prevent me from accepting the paper at this stage. They suggest, however, that a revised version might prove acceptable, if you can address their concerns. This will require additional experiments, as well as textual revisions, especially with regard to the criticisms of reviewers1 and 3. If you think that you can deal satisfactorily with the criticisms on revision, I would be pleased to see a revised manuscript. We would then return it to the reviewers.

Reviewer 1

Advance summary and potential significance to field

The paper by Diet, Senf and Neuhaus investigates the role of CXCR4 on neuronal differentiation in the vomeronasal organ (VNO). The combination of immunohistochemistry and the use of a conditional CXCR4 mouse line provides histological evidence that immature neurons found in the basal lineage showed a higher expression of CXCR4 compared to the apical lineage. Maturation of neuronal and non-neuronal cells in the VNO was affected in the conditional CXCR4 knockout mouse. Maturation of VNO stem cells was related to SOX2. The study revealed the possible contribution to neurogenesis of other transcription factors.

© 2025. Published by The Company of Biologists under the terms of the Creative Commons Attribution License (https://creativecommons.org/licenses/by/4.0/).

The complex cell transitions controlling neurogenesis in the VNO, as nicely illustrated in Fig. 1A, is limited and, this study provides interesting data on the role of CXCR4. The findings are relevant. I have two major comments on this work:

1) Quantification of immunohistochemistry images. The authors do not follow a standardized procedure to illustrate data. In some figures the authors should provide a more comprehensive evaluation of their results to strengthen their conclusions (indicated below). I suggest ending the paper with an updated Fig.1A to illustrate the findings of the current work and associate it to the discussion.

2) Complementing in silico findings. I do not see a clear association of Tfap2e in silico KO to the experimental data obtained. Could the authors complement Fig. 5A with images? It is an important point to validate the strategy and I do not see strong evidence for it in the way it is presented. Ideally, provide experimental evidence for other transcription factors identified in the in silico analysis.

Comments for the author

1) Quantification of immunohistochemistry images:
a) Fig.2A. I am not convinced by these images, which report a key finding to the study. In WT mice the intensity of the cells illustrated in the boxed region is comparable to the intensity of cells found outside of the boxed region. It looks like there is a gradient of SOX2 expression. In the CXCR4 cKO the cells are more intense outside than inside the boxed region. Is it true? What level of intensity is considered as a threshold to count a cell as positive? Would it be possible to show a double staining with a nuclei marker? It would be also necessary to display more than one histological section (ideally serially sectioned) to clearly show cell proliferation of SOX2 cells. A higher magnification is necessary to illustrate the cellular morphology. This type of information is necessary to convince the reader that there are more cells of a specific type in the cKO. The data of the box plot of Fig. 2B must be backed up with clearer images.
b) Fig. 2E. Same as in Fig. 2A. Would it be possible to show cell morphology in a higher magnification image and detail the intensity threshold for defining a positive vs a negative cell?
c) Fig. 3. This figure provides a quantification of cell intensity. It is necessary to normalize the data as function of the number of positive cells for a given marker, or, to indicate the change in positive cells in cKO mice. The authors are integrating all the fluorescence intensity, which contrasts to the strategy followed in Fig. 2. The authors must attempt to quantify cell densities, for example using the same methodology applied in Fig.4.

2) In silico KO of transcription factors. Please associate in silico data to experimental results in a single figure and provide more experimental data on other transcription factors.

<u>Reviewer 2</u>

Advance summary and potential significance to field

This study analyzes the role of the chemokine receptor CXCR4 in the replacement of sensory neurons in the vomeronasal organ of mice. Immunofluorescence staining and reporter gene expression under the CXCR4 promoter indicate high CXCR4 levels in the stem cell niche of the marginal zone and in derived neuronal cells of the sensory epithelium. Exploiting an existing single cell RNAseq dataset CXCR4 expression could be assigned to late stem cells (GBCs), neuronal precursors and basal sensory neurons. Conditional knockout of CXCR4 led to an increase of stem cell and proliferation markers (SOX2, MCM2) and a decrease of the neuroblast marker DCX indicating that CXCR4 is engaged in neurogenesis. Surprisingly, further analyses suggest enhanced differentiation of basal sensory neurons, a loss of non-neuronal horizontal basal cells, and an increase of sustentacular support cells. In silico gene perturbation using the recently established CellOracle phyton library for gene regulatory network construction provides additional evidence for the role of SOX2 and implicates a number of other transcription factors in GBC differentiation.

© 2025. Published by The Company of Biologists under the terms of the Creative Commons Attribution License (https://creativecommons.org/licenses/by/4.0/).

Overall these are interesting new findings with high relevance to the field, and I only have few comments and suggestions.

Comments for the author

1. The many abbreviations make reading difficult. This could be improved, at least a bit, if the respective abbreviations were included in Fig 1A.
2. The assignment of cell populations in Fig. 5A should also be applied to Fig. 2D, otherwise the description in the legend to Fig. 2D remains enigmatic. Moreover, the meaning of the arrows (size vs. direction) should be explained. If relevant, the authors should make sure that the resolution of these figures in the supplement is high enough to distinguish the arrows at higher magnification.
3. The sex of analyzed animals should be mentioned.
4. Line 112-117: Different numbers of analyzed regions and mice are quoted for "quantitative analysis" and "integrated densities". Please state clearly, to which data the respective numbers refer.
5. Line 129: "..three animals, and three independent qPCR runs…" Please clarify if, e.g., three independent technical qPCR replicates were performed for each animal (or, better, for each cDNA per animal).
6. Concerning the "delineation of the immature neuronal lineage" (Methods, line 138/139), it would be nice to get a flavor, how this was achieved, possibly by stating some of the key marker genes.
7. Line 183-187: The statement is somehow misleading. The missing tdTomato expression in the medial VNO is not indicating that "mature neurons move to the medial part". The sentence should be rephrased by first describing the expression in lateral neurons and by adding that the expression (or the lack of it) was analyzed at 8 weeks (i.e. 5 weeks after tamoxifen induction).
8. Line 247ff: The insertion explaining MCN2 make it hard to understand that the increase refers to MCM2-expressing cells. Please rephrase.
9. The authors should better discuss, how the increase in SOX2-positive GBCs fits to the increase of basal VSN in the CXCR4 ko VNO. As far as I understood, they assume the later to be a fate decision, but the discussion on this point is not well structured and confusing. Furthermore, if it is a fate decision, is there a decrease of apical VSN?
10. In the same context, I cannot follow the logic why Cxcr4 expression that "… extends into the basal lineage of iVSNs … indicates a distinct role…" (line 419/420). This should be elaborated in more detail. Similarly, the statement "indicates fate decision …showing an increased rate…" (line 421/422) should be revised (an "indication" cannot "show" something).
11. The discussion states "an increase in OMP expressing mVSNs" (line 414), as if there was an increase in cell numbers. This is somehow misleading, because the observation was just an increase of OMP expression level (by IF densitometry and qPCR). Please rephrase.

<u>Reviewer 3</u>

Advance summary and potential significance to field

In this manuscript by Dietz et al, they explore the role of CXCR4 in neuronal differentiation and tissue composition in the vomeronasal organ. They use conditional knockout of CXCR4 in GBCs to look at its role in proliferation and differentiation and conduct in silico analysis to identify additional transcription factors that may be involved in neurogenesis in this region. This gene has been studied in the migration of Gonadotropin-releasing hormone neurons in the developing VNO region in complete mutants but not in conditional knockout, nor has its role in the histogenesis of this region in adult (https://www.ncbi.nlm.nih.gov/pmc/articles/PMC3812513/). This study advances our understanding of the proliferative potential and histogenesis in the VNO, a tissue that is not well understood.

Overall, this study has potential to be interesting to the readers of this journal. However, there are significant issues around biological relevance and statistical analysis that must be addressed before recommendation for publication.

Comments for the author

© 2025. Published by The Company of Biologists under the terms of the Creative Commons Attribution License (https://creativecommons.org/licenses/by/4.0/).

Major revisions:
I have several major points that should be addressed before publication of the study:

1) The n numbers used for making the conclusions need to be increased. For most of the experiments n=3, which would indicate a single litter of animals. This is not sufficient to make a meaningful biological conclusion. A minimum of three biological replicates should be done and power calculations should be included.

2) It is not clear if the authors are comparing conditional knockouts to "WT" animals. Are these completely different strain wild type or litter matched genotyped wild types for the driver/floxed allele. If it is the former then this needs to be completely redone and reanalysed, as you can't compare across strains.

3) Some immunohistochemistry images (specifically Figure 2G, Figure 3, Figure 4C) are unconvincing. They state that this knockout affectes the basal population but show general diffuse staining and then do qPCR on the entire tissue. This is not sufficient to prove this phenotype. Further, while they back up IHC by qPCR data I am still unconvinced by a) whether this is real staining b) it doesn't appear to be cell specific to any cell population c) comparative fluorescent measurements across samples is not accurate or valid. There is no information in the methods as to whether staining is done on the same slide and imaged at identical settings before comparison. Even if so, the conclusions that can be drawn by intensity plots is limited and difficult to interpret.

Minor revisions:
Figure 1. The schematic is very helpful. Why not overlap tdTomato and antibody? It seems like the reporter is expressed more widely than the endogenous expression. What are bright spots in the image? Presumably microglia.

Figure 2. The authors chose to look at sox2 expression. However, it would have been better/more convincing if they used more than one marker as several have been identified (https://elifesciences.org/reviewed-preprints/97356).

It looks like there may be changed sox2+ morphology and/or organisation outside of the MZ. The authors should comment on this. Is it possible that proliferation of cells could be a global increase in cells and thus sox2+ increase. The authors should quantify number of nuclei in their MZ and normalise to Sox2+ cells in controls and cKO. n=3 not sufficiently powered to be conclusive.

What is the genotype of WT? If they are C57BL6/6J then wouldn't the proper control be Htr3a-Cre. However, Htr3a regulates sox2 expression (https://www.ncbi.nlm.nih.gov/pmc/articles/PMC9133002/). Is Htr3aCRE active earlier in the lineage and can it confound results?

How do they reconcile Cxcr4 expression vs the downregulation of DCX they observe across the whole tissue?

Figure 3. In 3A they say they see "exhibited a denser packaging of mVSNs". How did they measure this?

Why didn't they do qPCR for GÎ±i2?

Figure 4. 4C - What are they quantifying? I don't see any krt5+ cells in either image.

E,F - To me the morphology of the nuclei are different between the two. Is there a different density of cells which is why there is a difference in sox2+ cells?

G,H - Mcm2 clls in the CXCR4 looks like they are shifted to the MZ and not central like the WT. This is a very interesting phenotype that isn't well explained.

Figure 5. I'm not an expert on the validity of the in silico analysis, however the conclusions drawn from the present data are limited. It would have been more convincing if the authors validated

© 2025. Published by The Company of Biologists under the terms of the Creative Commons Attribution License (https://creativecommons.org/licenses/by/4.0/).

some of these markers in tissues in the conditional knockouts (as there is an Ap2 phenotype). Also, why did they do in silico knockout of tfap2e? Wouldn't it have made sense to do the cxcr4?

First revision

Author response to reviewers' comments

Specific responses to the reviewers

Reviewer #1

Comment: I suggest ending the paper with an updated Fig.1A to illustrate the findings of the current work and associate it to the discussion.

We would like to thank for the suggestion. We added an updated Fig. 1A as Fig.6 to summarize the results, the Figure is mentioned in the revised discussion.

Comment: 2) Complementing in silico findings. I do not see a clear association of Tfap2e in silico KO to the experimental data obtained. Could the authors complement Fig. 5A with images? It is an important point to validate the strategy and I do not see strong evidence for it in the way it is presented. Ideally, provide experimental evidence for other transcription factors identified in the in silico analysis.

In the revised version of the manuscript, we tried to improve the presentation of Figure 5. We changed the order of the figures, former Figure 5 is now Figure 4. We combined the in silico KO of Tfap2e, which shows delayed maturation of the basal lineage neurons, with our observation on Tfap2e expression in Cxcr4 cKO mice (now Fig. 4 B-D, former Fig. 3D-F). The observed increase in Tfap2e expression together with the enhanced presence of basal neurons fits well to the perturbation analysis showing that absence of Tfap2e slows basal neuron maturation. In addition, we thereby demonstrate the validity of the in silico approach. We included Atf5 (Fig.4E,F), since Atf5 is published to play a role in VNO neurogenesis. We also did Atf5 in-situ hybridization, but did not find a difference between WT and Cxcr4 cKO. Another transcription factor that is so far not described in VNO neurogenesis, but well known to influence differentiation of various epithelial cells, is Ovol2. This factor is also predicted to slow down basal neuron maturation, a hypothesis that may be worth testing in future. We added an IF staining for Ovol2, showing high expression level in VNO neurons of WT and Cxcr4-cKO mice (Fig. 4G). The final proof would of course be to generate e.g. Ovol2 cKO animals and investigate the cellular composition of the VNO. Unfortunately, we do not have KO's for so far uncharacterized transcription factors animals at hand. These experiments would therefore clearly be beyond the scope of the current manuscript. We also added a few more patterns of perturbation analysis outcomes (Fig.4I-N). With this approach we would like to demonstrate the power of CellOracle by screening the entire set of transcription factors in VNO neurogenesis and to identify interesting candidates that could be analyzed in upcoming studies.

SUGGESTIONS TO AUTHORs

1) Quantification of immunohistochemistry images:
a) Fig.2A. I am not convinced by these images, which report a key finding to the study. In WT mice the intensity of the cells illustrated in the boxed region is comparable to the intensity of cells found outside of the boxed region.

We added improved pictures. We indeed see bright SOX2-positive cells in the pictures, which are the sustentacular cells of the VNO. The cell can clearly be distinguished by localization and by analysis on the morphology of the nuclei, which is markedly different between sustentacular cells and basal cells in the marginal zone. A high magnification picture of the nuclear morphologies is presented in Fig.S2B We analyze these cells in more detail in the context of Figure 5, since these

© 2025. Published by The Company of Biologists under the terms of the Creative Commons Attribution License (https://creativecommons.org/licenses/by/4.0/).

are non-neuronal cells. The first approach (Fig.2) aimed at analyzing effects on neuronal cell lineage due to the known role of Cxcr4 in neurogenesis. For this, we wanted to quantify only the SOX2-positive basal stem cells in the marginal zone (boxed region).

It looks like there is a gradient of SOX2 expression. In the CXCR4 cKO the cells are more intense outside than inside the boxed region. Is it true?

This is indeed true, stem cells seem to express lower levels of SOX2 compared to sustentacular cell. Strong SOX2 expression in sustentacular cells is also present in the main olfactory epithelium, unfortunately so far without any experimental clue with regard to the function in these cells. SOX2 expression in sustentacular cells is analyzed and presented in Figure 5. Irrespective of the expression level in sustentacular cells, we wanted to analyze SOX2-positive stem cells in Figure 2. Both cell types can be clearly distinguished by nuclear staining (presented in the revised Fig. S2B)

What level of intensity is considered as a threshold to count a cell as positive? Would it be possible to show a double staining with a nuclei marker?

A double staining with Hoechst as nuclear marker is now included. We describe the threshold for counting cells as positive in the Materials and methods of the revised manuscript (lines 382-400): For cell counting, we determined the background staining intensity in cells that do not express the protein of interest based on visual inspection. We then counted cells which have intensity value which are minimum five times the background value as positive.

It would be also necessary to display more than one histological section (ideally serially sectioned) to clearly show cell proliferation of SOX2 cells. A higher magnification is necessary to illustrate the cellular morphology. This type of information is necessary to convince the reader that there are more cells of a specific type in the cKO. The data of the box plot of Fig. 2B must be backed up with clearer images.

We tried to include better images. We would also like to mention that we counted cells on single confocal sections, but used stacks of confocal section for better visualization of the cells. We now add a co-staining of SOX2 with MCM2 in higher magnification (Fig.S2A). Co- labeling showed that only few SOX2+ cells are proliferating. So we see more proliferating cells and more SOX2 cells, but highest level of SOX2 expression does not seem to occur in proliferating cells. This is not unexpected, as high expression level of SOX2 maintain stem cells of the central nervous system in a slowly self-renewing undifferentiated state and proliferation is accompanied by decreased SOX2 expression (statement also included in the revised manuscript lines 156-159).

b) Fig. 2E. Same as in Fig. 2A. Would it be possible to show cell morphology in a higher magnification image and detail the intensity threshold for defining a positive vs a negative cell?

The detailed description of the intensity threshold is described in the methods (see also previous answer). A higher magnification picture is now included.

c) Fig. 3. This figure provides a quantification of cell intensity. It is necessary to normalize the data as function of the number of positive cells for a given marker, or, to indicate the change in positive cells in cKO mice. The authors are integrating all the fluorescence intensity, which contrasts to the strategy followed in Fig. 2. The authors must attempt to quantify cell densities, for example using the same methodology applied in Fig.4.

Unfortunately, it is very difficult to unambiguously determine cell numbers for some of the stainings. Gnai is nearly exclusively localized in the microvilli of the sensory neurons. The resolution of the light microscopy pictures does not allow to determine the number of cells from the microvillar layer due to lack of clear separation between single cells. In a similar manner, the staining pattern of NCAM2 does not allow to clearly count cells. Similarly, OMP labeled cells show dense packing which precludes to clear identify single cells. Gnao positive cells were counted and the results are added (Fig.3F).

2) In silico KO of transcription factors. Please associate in silico data to experimental results in a

© 2025. Published by The Company of Biologists under the terms of the Creative Commons Attribution License (https://creativecommons.org/licenses/by/4.0/).

single figure and provide more experimental data on other transcription factors.

We would like to thank for the helpful suggestion and associated the experimental results on Tfap2e to the in silico data (now Fig.4). In addition, we stained for two more transcription factors with interesting results in the in-silico analysis. We stained mRNA of Atf5, which is known to affect neurogenesis in the VNO, by in situ hybridization (Fig.4F). We did not try to quantify differences between genotypes, as all stained sections essentially looked the same. ATF5 antibody staining was not successful in our hands. We also added staining of another transcription factor (OVOL2), which has an interesting prediction result and showed robust expression in the neurons of the VNO. The stainings were not different between WT and Cxcr4-cKO mice, indicating that also this transcription factor does maybe not play a role in Cxcr4 signaling (Fig.4H,I). Final proof for a role of the transcription factors analyzed anyhow would require analysis of specific knock out mice, which we do not have in the lab. We nevertheless think the in silico analysis may be helpful for other research projects in future. Even if is "only" in silico, it still offers biological insight into VSN neurogenesis.

Reviewer 2: SUGGESTIONS TO AUTHORS

1. The many abbreviations make reading difficult. This could be improved, at least a bit, if the respective abbreviations were included in Fig 1A.

We agree with your impression that it is easier to read with lesser abbreviations. We removed all abbreviations except VNO and VSN. We hope this improves the overall impression and readability.

2. The assignment of cell populations in Fig. 5A should also be applied to Fig. 2D, otherwise the description in the legend to Fig. 2D remains enigmatic. Moreover, the meaning of the arrows (size vs. direction) should be explained. If relevant, the authors should make sure that the resolution of these figures in the supplement is high enough to distinguish the arrows at higher magnification.

We assigned cell populations in Fig. 2D (now Fig. 2E) and we explain the meaning of the arrows in the legend for Fig. 4. The resolution is high enough in the tiff files uploaded in the revision process. We would like to apologize if the resolution was insufficient in the merged pdf of the initial submission.

3. The sex of analyzed animals should be mentioned.

We used mice of both sexes since we did not observe any differences between sexes in the parameters analyzed in this study and to fulfill the 3R requirements in our facility. We state this in the Methods part of the revised manuscript.

4. Line 112-117: Different numbers of analyzed regions and mice are quoted for "quantitative analysis" and "integrated densities". Please state clearly, to which data the respective numbers refer.

We state the numbers in the figure legends of the revised manuscript.

5. Line 129: "..three animals, and three independent qPCR runs…" Please clarify if, e.g., three independent technical qPCR replicates were performed for each animal (or, better, for each cDNA per animal).

We clarified the statement according to your suggestion (now lines 412-413).

6. Concerning the "delineation of the immature neuronal lineage" (Methods, line 138/139), it would be nice to get a flavor, how this was achieved, possibly by stating some of the key marker genes.

We listed the used marker genes, which were taken from published paper, in the Methods part (439-445).

© 2025. Published by The Company of Biologists under the terms of the Creative Commons Attribution License (https://creativecommons.org/licenses/by/4.0/).

7. Line 183-187: The statement is somehow misleading. The missing tdTomato expression in the medial VNO is not indicating that "mature neurons move to the medial part". The sentence should be rephrased by first describing the expression in lateral neurons and by adding that the expression (or the lack of it) was analyzed at 8 weeks (i.e. 5 weeks after tamoxifen induction).

We rephrased the statement (now lines 99-106).

8. Line 247ff: The insertion explaining MCN2 make it hard to understand that the increase refers to MCM2-expressing cells. Please rephrase.

We rephrased this sentence, now lines 151-159.

9. The authors should better discuss, how the increase in SOX2-positive GBCs fits to the increase of basal VSN in the CXCR4 ko VNO. As far as I understood, they assume the later to be a fate decision, but the discussion on this point is not well structured and confusing. Furthermore, if it is a fate decision, is there a decrease of apical VSN?

We rephrased this part of the discussion (lines 288-290). We also added data on apical neurons and state that the apical lineage is likely not affected by CXR4 cKO.

10. In the same context, I cannot follow the logic why Cxcr4 expression that "... extends into the basal lineage of iVSNs ... indicates a distinct role..." (line 419/420). This should be elaborated in more detail. Similarly, the statement "indicates fate decision ...showing an increased rate..." (line 421/422) should be revised (an "indication" cannot "show" something).

We rephrased this statement in the revised manuscript (now lines 294-301).

11. The discussion states "an increase in OMP expressing mVSNs" (line 414), as if there was an increase in cell numbers. This is somehow misleading, because the observation was just an increase of OMP expression level (by IF densitometry and qPCR). Please rephrase.

We rephrased this statement (line 288-290).

Reviewer 3: SUMMARY OF THE ADVANCE MADE IN THIS PAPER AND ITS POTENTIAL SIGNIFICANCE TO THE FIELD SUGGESTIONS TO AUTHORS

Major revisions: I have several major points that should be addressed before publication of the study:

1) The n numbers used for making the conclusions need to be increased. For most of the experiments n=3, which would indicate a single litter of animals. This is not sufficient to make a meaningful biological conclusion. A minimum of three biological replicates should be done and power calculations should be included.

We did not used single litters for our analysis, but included mice from different litters. We increased the number of biological replicates in the PCR experiments. We also analyzed more animals to increase the numbers in the IF experiments. We also did power calculations. For the effect strength and p-values that we measured, we obtained power values of 81%-99%.
We also changed the labeling of the significance levels to present three levels ($p<0.05$, **$p<0.01$, ***$p<0.001$).*

2) It is not clear if the authors are comparing conditional knockouts to "WT" animals. Are these completely different strain wild type or litter matched genotyped wild types for the driver/floxed allele. If it is the former then this needs to be completely redone and reanalysed, as you can't compare across strains.

The background strain in all cases is C57Bl6/J, the comparison of mice is therefore possible. In

© 2025. Published by The Company of Biologists under the terms of the Creative Commons Attribution License (https://creativecommons.org/licenses/by/4.0/).

addition, we analyzed Gnao expression in HTR3a-Cre mice (driver line, C57Bl6/J background) and found no difference compared to WT (C57Bl6/J) animals (revised Fig. S3D). Also analyzed SOX2/MCM2 expression in 5HT3-Cre mice and found no difference to WT either. (Fig.S2A,B).

3) Some immunohistochemistry images (specifically Figure 2G, Figure 3, Figure 4C) are unconvincing. They state that this knockout affectes the basal population but show general diffuse staining and then do qPCR on the entire tissue. This is not sufficient to prove this phenotype. Further, while they back up IHC by qPCR data I am still unconvinced by a) whether this is real staining b) it doesn't appear to be cell specific to any cell population c) comparative fluorescent measurements across samples is not accurate or valid. There is no information in the methods as to whether staining is done on the same slide and imaged at identical settings before comparison. Even if so, the conclusions that can be drawn by intensity plots is limited and difficult to interpret.

We agree that some picture can be improved. We tried to provide more convincing data in the revised manuscript. We replaced Fig.2g (now Fig. 2K) by a different picture. Moreover, we accompany the stainings with a feature plot, showing that DCX is expressed by cells of both neuronal lineages (Fig.2I, Fig.S2C,D). DCX is a clear neuronal protein (Fig.S2C,D), mRNA detected by PCR in the whole tissues can therefore interpreted as being derived from the neuronal cells. Similarly, we replaced pictures in Figure 3 and show additional pictures in Fig. S3. We also provide feature plots of basal VSN markers (Fig. 3C, Fig.S3E). We also show feature plots of apical markers (Fig.3I,K) together with corresponding PCR results (Fig. 3H). We state, that while Gnai2 expression distinguishes apical from basal neurons (Fig.3C), Gnai2 is also expressed by other cells in the VNO (Fig.S3E). PCR results from Gnai2 are shown in the revised version (Fig. 3H), but cannot be interpreted as showing increased expression in apical neurons due to the broad expression pattern. We also replaced Fig.4C for another picture (now Fig.5C and Fig. S5).

Minor revisions:

Figure 1. The schematic is very helpful. Why not overlap tdTomato and antibody? It seems like the reporter is expressed more widely than the endogenous expression. What are bright spots in the image? Presumably microglia.

We did co-staining with Iba1 (marker for macrophages) and Icam1 (marker for blood vessel endothelial cells) to show that addition tdTomato expression occurs in these structures. Cxcr4 is not specific to neurons, and it is well known that both structures are Cxcr4-positive. The staining is presented in Fig.1D and Fig.S1A. The fact that the structures are not labeled by the antibody show that the expression level of CXCR4 in these structures is not strong enough to be seen by normal IF staining. CXCR4 staining in these structures is usually done by using amplification kits, which did not use to stain CXCR4 in the VNO.

Figure 2. The authors chose to look at sox2 expression. However, it would have been better/more convincing if they used more than one marker as several have been identified ().

We added the analysis of Ascl1, Neurog1 and Neurod1 by quantitative PCR. Unfortunately, stainings for Ascl1 and Neurod1 with established antibodies did not result in convincing staining patterns in the adult VNO. All markers are specific for stem cells in the VNO, which we state by citation of the mentioned article. PCR is therefore the only direct way to analyze expression of these markers, since we do not have Cre mice for lineage tracing.

It looks like there may be changed sox2+ morphology and/or organisation outside of the MZ. The authors should comment on this. Is it possible that proliferation of cells could be a global increase in cells and thus sox2+ increase. The authors should quantify number of nuclei in their MZ and normalise to Sox2+ cells in controls and cKO. n=3 not sufficiently powered to be conclusive.

There were indeed changes in SOX2 expression in apical sustentacular cells. These cells were analyzed and quantified in the revised Fig.5 (initial submission Fig.4). All sustentacular cells were SOX2-positive. In the marginal zone, not all cells were SOX2-positive. Unfortunately, the marginal

© 2025. Published by The Company of Biologists under the terms of the Creative Commons Attribution License (https://creativecommons.org/licenses/by/4.0/).

zone has no clear demarcation with respect to the medial border. It is therefore not possible to unambiguously count all cells in the marginal zone. We counted cell numbers in 2 more animals (now n=5).

What is the genotype of WT? If they are C57BL6/6J then wouldn't the proper control be Htr3a-Cre. However, Htr3a regulates sox2 expression (https://www.ncbi.nlm.nih.gov/pmc/articles/PMC9133002/). Is Htr3aCRE active earlier in the lineage and can it confound results?

As mentioned above, the background strain in all cases is C57Bl6/J. We analyzed Gnao expression in HTR3a-Cre mice (driver line, C57Bl6/J background) and did not find any difference compared to WT animals. SOX2 is essential for embryonic development from the first stages of embryo formation. It is expressed in the inner cell mass of the mouse embryo and homozygous null mutations results in the arrest of embryonic development just after the embryo implants in the uterus. 5-HT3A expression was detected at 10 days post coitus (dpc) in neural crest derivatives of transgenic Htr3a-EGFP reporter mice, (Ritter KE, Southard- Smith EM. Front Neurosci. 2017 Jan 6;10:592. doi:10.3389/fnins.2016.00592.). 5-HT3A is therefore active later and is unlikely to confound Sox2 expression. Moreover, as it is a transgenic strain, Htr3a expression is not affected in the driver line.

How do they reconcile Cxcr4 expression vs the downregulation of DCX they observe across the whole tissue?

Dcx is expressed in immature neurons, whereas Cxcr4 is expressed earlier (can be seen in Fig.2D versus Fig.2I). The difference in Dcx expression in the neuronal lineage therefore could be a consequence of Cxcr4 expression in the progenitor cells. Dcx is a purely neuronal protein (Fig.S2C,D), analysis of the whole tissue therefore recapitulates expression in VSNs. There are autonomic nerve endings surrounding the blood vessel of the VNO, but due to absence of their cell bodies in the preparation are unlikely to contribute markedly to the total amount of Dcx mRNA.

Figure 3. In 3A they say they see "exhibited a denser packaging of mVSNs". How did they measure this?

We did not measure this, we only provide a qualitative impression due to lesser non-labeled space between the neurons.

Why didn't they do qPCR for Gαi2?

We did do qPCR analysis of Gnai2. We initially decided against showing the data, since Gnai2 is expressed by multiple cell types in the VNO preparation. It is therefore not possible to infer from total mRNA in the tissue to the amount of apical neurons. We now added the PCR result (Fig.3H), together with the PCR result of a gene that is very specific for apical VSNs (Pcdh7). The broad expression pattern can be seen in the feature plot for Gnai2 (now Fig.S3F).

Figure 4. 4C - What are they quantifying? I don't see any krt5+ cells in either image.

We tried to provide better pictures for Krt5 (Fig.5C and Fig.S5C) and hope the cells become visible in the revised figures. The horizontal basal cells in the VNO are flat, relatively small cells, their visibility may be improved in higher magnification pictures provided.

E,F - To me the morphology of the nuclei are different between the two. Is there a different density of cells which is why there is a difference in sox2+ cells?
It is true that the morphologies of the nuclei are different, providing additional evidence for an effect of the Cxcr4 cKO. If we count more SOX2-positive nuclei fer 100 nm in the apical cell layer, this per definition shows an increased densitiy. G,H - Mcm2 clls in the CXCR4 looks like they are shifted to the MZ and not central like the WT. This is a very interesting phenotype that isn't well explained.

It is an interesting observation. We are not sure if it is a shift of the cells. It may also be, that we

© 2025. Published by The Company of Biologists under the terms of the Creative Commons Attribution License (https://creativecommons.org/licenses/by/4.0/).

have Mcm2-positive cells in the marginal zone, which increased in Cxcr4 cKO animals (see also Fig. 2F). We have lesser Mmc2 positive nuclei in the sustentacular cell layer, which may be cells derived from the cells in the marginal zone or it may be a shift of the cells. Answering this interesting question would clearly require more experiments. In addition, a conclusive explanation for the observed distribution of MCM2 cells also likely has to await further experimentation.

Figure 5. I'm not an expert on the validity of the in silico analysis, however the conclusions drawn from the present data are limited. It would have been more convincing if the authors validated some of these markers in tissues in the conditional knockouts (as there is an Ap2 phenotype). Also, why did they do in silico knockout of tfap2e? Wouldn't it have made sense to do the cxcr4?

The in-silico approach with CellOracle builds custom gene regulatory network models that are specifically designed to simulate shifts in cell identity following transcription factor (TF) perturbation. This helps to provide a systematic interpretation of context-dependent TF function in regulating cell identities. Results in haematopoiesis and in zebrafish development showed that CellOracle can be used to infer and interpret cell-type-specific GRN configurations at high resolution, enabling mechanistic insights into the regulation of cell identity. (Kamimoto, K., Stringa, B., Hoffmann, C.M. et al. Dissecting cell identity via network inference and in silico gene perturbation. Nature ***614****, 742-751 (2023). https://doi.org/10.1038/s41586-022-05688-9). As Cxcr4 is not a transcription factor, it cannot be analyzed with this approach. The Ap2e phentotype is published, we presented the data to show the validity of our VNO gene regulatory network model and as Ap2e expression was altered in Cxcr4 cKO mice. A real experimental validation would be the analysis of an unkown trancription factor together with a KO approach. However, this would be beyond the scope of this manuscript. We did however analyze the expression of ATF5 and Ovol2 in the adult VNO.*

Second decision letter

MS ID#: JOCES/2024/263451

MS TITLE: Stem cell expressed CXCR4 regulates tissue composition in the vomeronasal organ

AUTHORS: André Dietz; Katja Senf; Eva M. Neuhaus
ARTICLE TYPE: Research Article

We have now reached a decision on the above manuscript.

To see the reviewers' reports and a copy of this decision letter, please go to:

As you will see, the reviewers have now given favourable reviews of your manuscript, but two of them raised some critical points that will require some amendments to your manuscript. I hope that you will be able to carry these out because I would like to be able to accept your paper.

Reviewer 1

Advance summary and potential significance to field

The authors have satisfactorily addressed my comments. The paper has increased in readability and the quality of the figures has improved. I particularly like the discussion and new Figure 6, which conveys a clear message of the mechanism proposed.

I still have some minor comments all related to images. Some of them might come from the low resolution of the pdf but I would like to express them, especially for publication purposes.

Specifically:

© 2025. Published by The Company of Biologists under the terms of the Creative Commons Attribution License (https://creativecommons.org/licenses/by/4.0/).

1) Fig 1B appears in a low quality. I cannot distinguish individual cells.
2) Line 98, "lateral cells showing a neuronal morphology". Please indicate in image.
3) Quantification of Sox2+ cells. I acknowledge the explanation in the methods section, however, a good complement could be an image illustrating a positive (counted) and a negative (non-counted) cell. For example, in Fig. S2A the authors only show nuclei. Why do not showing also the Sox2 channel to distinguish between Sox + and - cells?
4) Line 140. "significant increase". Why do not state the increase, i.e. threefold?
5) Fig. 2K. Low quality. Please check.
6) Fig. 4E. Low quality. Please use dotted white lines to illustrate the region indicated.
7) Fig. 6. Why do not graphically show the KO for Cxcr4? It is stated in the legend, but it could be presented opposed to Fig. 1A, for example by changing a colour, or, including a title.

I want to thank the authors for their work, and I recommend this paper for publication.

Reviewer 2

Advance summary and potential significance to field

The revised manuscript appears greatly improved and all points raised by the reviewer have been dealt with.

The authors should, however, give it another round or proof-reading to eliminate remaining typos and grammar issues, e.g. line 157 " (a) high expression level(s) of SOX2 maintain(s)", line 210 "has been described to affect(s)", line 215 "the observed increase(d) in the number".

Moreover, in Line 118, I do not understand what is meant by "such as demonstrated for Cxcr4", and in line 213 "using CellOracle also revealed" I would delete "also" and replace "revealed" by "suggested", "indicated", or similar.

Reviewer 3

Advance summary and potential significance to field

The authors have mostly addressed my concerns and I can now recommend publication.

Second revision

Author response to reviewers' comments

Dear Dr. Hill,

Please find attached the second revision of our manuscript "Stem cell expressed CXCR4 regulates tissue composition in the vomeronasal organ", in which we carefully addressed the remaining criticisms raised by the reviewers. Thus, we hope our manuscript will now be regarded suitable for publication in *Journal of Cell Science*. We thank you for your time and consideration and look forward to your response,
Eva Neuhaus

Comments from the Reviewers:
Reviewer 1:

1) Fig 1B appears in a low quality. I cannot distinguish individual cells.
We changed the picture and present the new staining in green to increase visibility. We hope the resolution is now sufficient.
2) Line 98, "lateral cells showing a neuronal morphology". Please indicate in image.

© 2025. Published by The Company of Biologists under the terms of the Creative Commons Attribution License (https://creativecommons.org/licenses/by/4.0/).

We indicated the lateral cells with white arrows in the revised version.
3) Quantification of Sox2+ cells. I acknowledge the explanation in the methods section, however, a good complement could be an image illustrating a positive (counted) and a negative (non-counted) cell. For example, in Fig. S2A the authors only show nuclei. Why do not showing also the Sox2 channel to distinguish between Sox + and - cells?
We replaced Fig.S2A by an overlay image, as suggested.
4) Line 140. "significant increase". Why do not state the increase, i.e. threefold?
We state a 2.5fold increase in the revised version.
5) Fig. 2K. Low quality. Please check.
May be due to the pdf conversion, the quality of the tiff file is fine.
6) Fig. 4E. Low quality. Please use dotted white lines to illustrate the region indicated.
May be due to the pdf conversion, we added dotted lines.
7) Fig. 6. Why do not graphically show the KO for Cxcr4? It is stated in the legend, but it could be presented opposed to Fig. 1A, for example by changing a colour, or, including a title.
We replace the graphical summary to show the results of the Cxcr4 knockout in the revised Figure 6.
Reviewer 2: The revised manuscript appears greatly improved and all points raised by the reviewer have been dealt with.
We apologize for the mistakes; we corrected the mistakes as suggested and went through another round of proof-reading.

Third decision letter

MS ID#: JOCES/2024/263451

MS TITLE: Stem cell expressed CXCR4 regulates tissue composition in the vomeronasal organ

AUTHORS: André Dietz; Katja Senf; Eva M. Neuhaus
ARTICLE TYPE: Research Article

I am happy to tell you that your manuscript has been accepted for publication in Journal of Cell Science, pending standard publication integrity checks.

© 2025. Published by The Company of Biologists under the terms of the Creative Commons Attribution License (https://creativecommons.org/licenses/by/4.0/).
